# An Expertise Transfer Framework For Autonomous Surgical Assistance

**DOI:** 10.1002/advs.78020

**Published:** 2026-09-28

**Authors:** Yuan Gao, Guangdi Chu, Wei Jiao, Yuchuan Zhong, Hang Yuan, Chengjun Li, Jinhua Li, Shuxin Wang, Bo Guan, Jianchang Zhao, Lizhi Pan, Haitao Niu, Jianmin Li

**Affiliations:** ^1^ Institute of Medical Robotics and Intelligent Systems Tianjin University Tianjin China; ^2^ Academy for Mechanical Discipline Studies Nanyang Institute of Technology Nanyang China; ^3^ Key Laboratory of Mechanism Theory and Equipment Design of Ministry of Education, School of Mechanical Engineering Tianjin University Tianjin China; ^4^ Department of Urology The Affiliated Hospital of Qingdao University Qingdao China; ^5^ Department of Hepatobiliary and Pancreatic Surgery Zhongshan Torch Development Zone People's Hospital Zhongshan China; ^6^ Chongqing University Chongqing China

**Keywords:** autonomous surgery, expertise transfer, minimally invasive surgery, surgical scene understanding, trustworthy autonomy

## Abstract

Autonomous surgery holds the potential to transform operative practice by enhancing procedural safety and consistency. While substantial progress has been made on episodic sub‐tasks, achieving autonomy for long‐horizon tasks that span the entire procedure remains challenging. Current logic‐based heuristics and end‐to‐end learning approaches, though effective for short‐term actions, struggle to adapt to the evolving dynamics of complete surgical workflows. To address this gap, an expertise transfer framework is introduced to achieve procedure‐spanning autonomous view assistance by emulating expert logic. Specifically, the framework instantiates situational awareness via a novel hierarchical granularity perception network that jointly captures surgical workflow and fine‐grained instrument‐tissue interactions; models attention allocation for inferring assistance needs by systematically characterizing surgeon visual attention patterns; and encodes decision‐making logic for actionable behaviors by formalizing expert assistance policies as a heterogeneous knowledge graph. Equipped with this architecture, the proposed framework successfully enables autonomous view assistance in vivo animal trials throughout complete nephrectomy procedures, while demonstrating improved visualization quality compared to human assistance. This expertise transfer paradigm provides a promising pathway toward trustworthy autonomy in clinical applications, advancing the shift toward procedure‐spanning autonomous workflows.

## Graphical abstract

This study introduces an expertise transfer framework for procedure‐spanning autonomous surgical assistance. By emulating expert logic through hierarchical perception, attention modeling, and knowledge graph‐based decision‐making, the system provides near‐expert surgical view assistance. Successfully validated in vivo animal trials during complete nephrectomies, this approach enhances visualization quality and offers a promising pathway toward long‐horizon surgical autonomy.

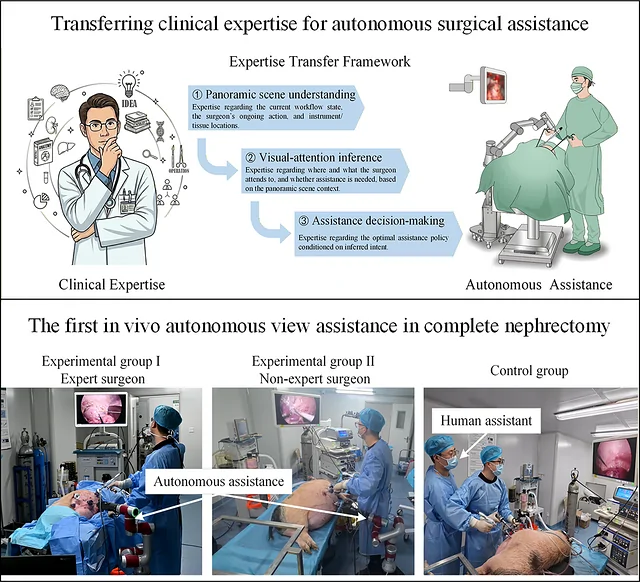

## 1 Introduction

Minimally invasive surgery (MIS) has revolutionized modern medicine, offering reduced trauma and accelerated recovery for millions of patients annually worldwide [1, 2, 3]. Building on this success, autonomous surgical systems offer the potential to ensure consistent, high‐quality support, thereby improving the clinical outcomes of MIS. Significant progress has been demonstrated in vivo animal models across diverse autonomous surgical subtasks, including suturing [4], cystic duct dissection [5], gauze retrieval [6], and targeted retinal injections [7]. These pioneering contributions demonstrate exceptional proficiency in achieving autonomy for episodic sub‐tasks within short operational windows. However, research addressing continuous autonomy for tasks that span the entire surgical procedure remains sparse.

Surgical assistance represents a quintessential example of such a continuous task, permeating virtually every MIS procedure. Due to the intrinsic constraints of keyhole access and indirect visualization, the primary surgeon relies heavily on coordinated assistance [8, 9]. Among support functions, intraoperative field of view (FoV) management is paramount. Proficient view assistance is critical for ensuring a stable surgical view, potentially reducing operative time and lowering the risk of intraoperative complications [10, 11, 12]. Beyond this crucial clinical significance, the essence of this task shares the same underlying logic as autonomous surgery. Specifically, effective view assistance necessitates a comprehensive understanding of the complex surgical scene and robust decision‐making. These capabilities constitute the core engine required for achieving autonomy throughout the entire surgical procedure [13, 14]. Driven by this rationale, we position autonomous view assistance as an entry point to advance progress toward autonomy throughout the entire surgical procedure.

Beyond advancing full‐procedure autonomy, autonomous assistance itself bears profound clinical significance. Current clinical assistance relies predominantly on manual effort and limited robotic support, yet both approaches impose intrinsic limitations. Manual assistance (Figure 1a) is plagued by fatigue‐induced instability and physical interference with the surgeon. These factors disrupt surgical continuity and pose potential safety risks [15, 16]. Moreover, cultivating a competent assistant entails not only a steep individual learning curve but also a prolonged collaboration curve [1, 17]. To address these human constraints and optimize intraoperative teamwork, researchers have conceptualized diverse robotic surgical assistants, encompassing various support roles such as endoscope manipulation and multimodal scrub nursing [18, 19]. While robotic systems (Figure 1b) mitigate these physical constraints, current commercial solutions predominantly rely on master‐slave operation. This requires the surgeon to not only perform the surgical procedure but also manually control the robotic system via foot pedals or head sensors [20, 21]. Consequently, it significantly increases workload and diverts attentional resources from core surgical tasks [22, 23]. In contrast, autonomous view assistance eliminates the reliance on manual holding and master‐slave control, ensuring stable visualization while restoring the surgeon's full focus to the procedure itself. Consequently, the pursuit of assistance autonomy holds profound clinical significance.

**FIGURE 1:**
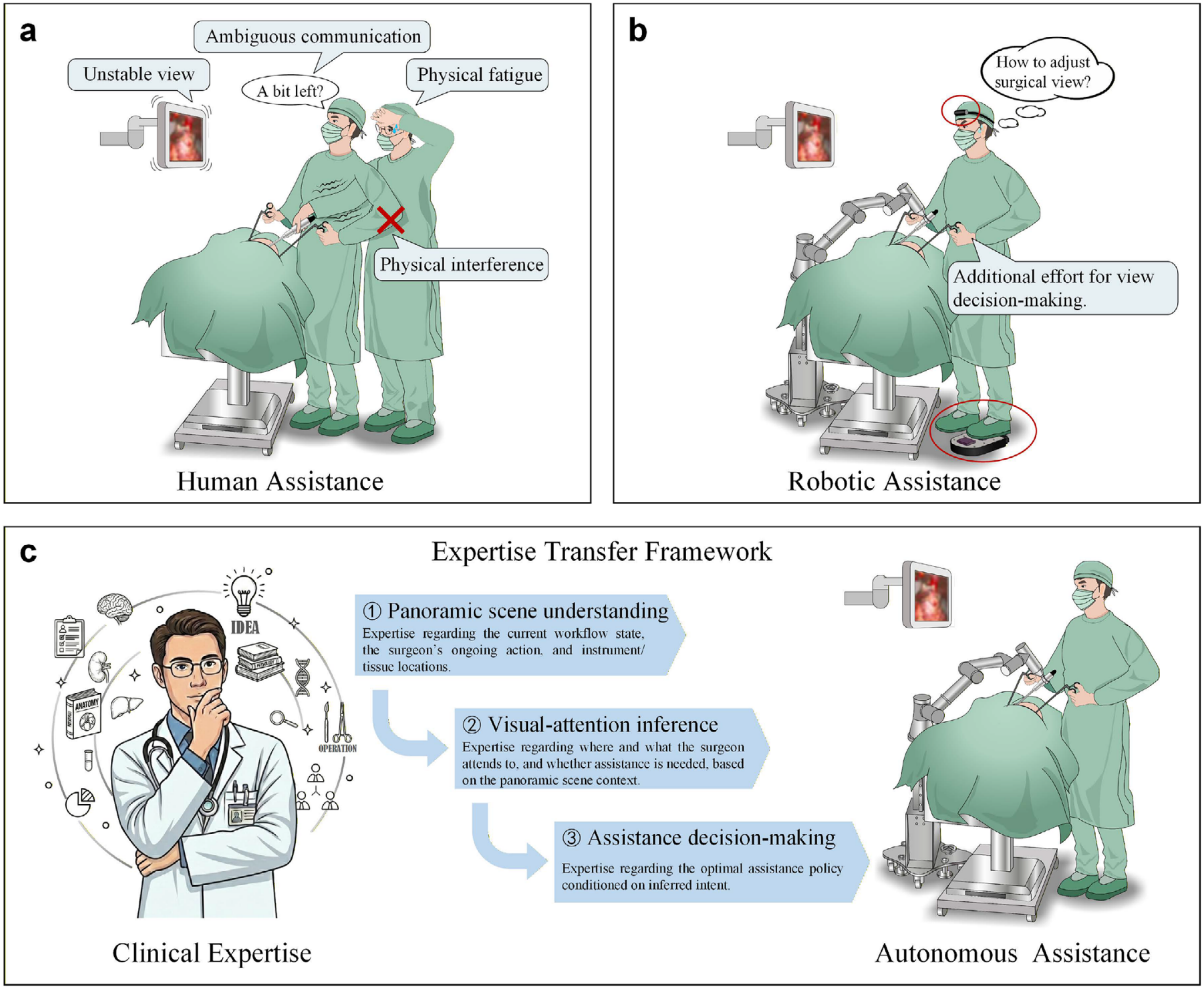
Comparison of human assistance, robotic assistance, and our autonomous assistance. (a) Human assistance is limited by unstable views, ambiguous verbal communication, physical fatigue, potentially increasing procedure time and surgical risk. (b) Robotic assistance often relies on surgeon‐operated teleoperation interfaces (e.g., foot pedals and head‐motion sensors; highlighted in red), requiring the surgeon to operate while making additional view decisions, which can interrupt workflow and increase overhead. (c) Our expertise transfer framework enables autonomous assistance by learning three levels of expert capabilities: (1) panoramic scene understanding (workflow state, ongoing action, and instrument/tissue locations); (2) visual‐attention inference (where/what the surgeon attends to and whether assistance is needed, based on scene context); and (3) assistance decision‐making (assistance policy conditioned on inferred intent). Together, our framework enables successful autonomous view assistance throughout the surgical workflow in vivo trials.

Motivated by this clinical significance, researchers have actively explored solutions for autonomous view assistance. Existing approaches generally fall into two categories: rule‐based and learning‐based. Rule‐based methods typically use deep neural networks to localize surgical instruments and then apply predefined heuristics (e.g., instrument‐centering strategies) to adjust the FoV [24, 25, 26, 27]. Conversely, learning‐based methods attempt to derive FoV assistance policies directly from surgical videos via end‐to‐end imitation learning or learning from demonstration [28, 29, 30]. However, rule‐based systems are limited by static logic, while learning‐based models suffer from stability issues like catastrophic forgetting. These approaches lack the temporal robustness required to adapt to the continuous evolution of a full surgical procedure. Moreover, the clinical environment imposes significantly higher demands for interpretability [31, 32, 33]. A transparent assistance process not only guarantees safe behavioral boundaries but also enables the surgeon to anticipate collaborative actions, allowing them to operate with confidence. In contrast, opaque decision logic compels the surgeon to hesitate during maneuvers and expend extra effort adapting to the autonomous system, thereby compromising surgical efficiency and safety [34, 35]. These challenges render the opaque methods that excel in general domains unsuitable for clinical deployment, creating a critical bottleneck in evolving from episodic sub‐tasks to full‐procedure autonomy. As a result, validation of autonomous view assistance throughout the entire surgical procedure under in vivo settings remains limited.

To overcome these challenges, we turn to clinical practice for inspiration. Experienced human assistants exhibit a distinct multi‐level decision‐making process: they comprehensively perceive the multi‐granular surgical scene (including surgeon actions, instrument‐tissue dynamics, and procedural evolution); simultaneously infer the surgeon's visual attention and assistance needs; and ultimately leverage this expertise to make appropriate assistance decisions. Inspired by this, we propose an expertise transfer framework for autonomous surgical assistance, as shown in Figure 1c. By systematically deconstructing and reproducing this expert reasoning, we aim to construct a trustworthy autonomous assistance framework optimized for procedure‐spanning clinical tasks.

Specifically, our work is materialized as a three‐level expertise transfer paradigm. First, we develop a unified hierarchical granularity perception network (HGPNet) that transfers expert‐level panoramic awareness to the robot, enabling holistic surgical scene understanding from macroscopic phase and step recognition to microscopic instrument–action–tissue triplet recognition and pixel‐level segmentation. This holistic approach overcomes the fragmentation of existing single‐task methods, providing a comprehensive informational basis for decision‐making. Second, we systematically reveal the implicit correlation between the surgeon's intraoperative visual attention and the dynamic surgical scene, filling a critical gap in the field. By distilling this expertise into a mathematically formulated attention prior model, we transfer the expert's ability to infer the surgeon's auxiliary intentions to the robot. Finally, based on this dual awareness, we formalize the expert's decision logic into a heterogeneous knowledge graph to derive dynamic decision policies. This achieves the transformation from tacit expert knowledge to explicit robotic execution strategies, ensuring that every autonomous assistance action is semantically traceable to specific scene states, thereby meeting the stringent safety and explainability requirements of clinical environments.

We evaluated the clinical translational potential of our framework through in vivo porcine laparoscopic nephrectomy (LN) experiments, a technically demanding procedure requiring precise dissection and vascular control. In collaboration with surgeons of varying experience levels, our framework delivered autonomous view assistance throughout the entire surgical workflow. Comparative results further demonstrate superior FoV stability and visual content quality relative to human assistants. To the best of our knowledge, this work provides the first in vivo demonstration of fully autonomous surgical view assistance for a complete soft‐tissue procedure, beyond simulation‐based evaluations and fragmented subtasks in simplified settings typical of prior studies. Our framework also demonstrated cross‐procedure generalization on simulated laparoscopic cholecystectomy (LC). Taken together, these results suggest that the expertise transfer paradigm can handle the complexity and diversity of clinical applications, providing a promising pathway to advance the field toward fully autonomous surgery.

## 2 Results

In the following sections, we report the transfer results of the multi‐level expertise that underpins the framework. We first evaluate HGPNet under single‐task, cross‐dataset, and joint multi‐task settings. This component constitutes the transfer of comprehensive situational awareness and provides the perceptual basis for downstream decision making. Leveraging these hierarchical granularity outputs together with eye‐tracking data, we then present results that systematically reveal surgeons' visual‐attention behaviors across surgical contexts as an operational proxy for collaborative intent. We further validated the intent‐to‐action transfer, that is, end‐to‐end autonomous surgical assistance, in vivo trials throughout complete LN procedures. We additionally assessed robustness when collaborating with surgeons of different experience levels and quantified performance against a human assistant. Finally, we evaluated cross‐procedure generalization beyond LN by deploying our framework in LC.

### 2.1 Hierarchical Granularity Surgical Scene Understanding

As illustrated in Figure 2a, HGPNet simultaneously outputs surgical phase, surgical step, surgical action triplets, and surgical scene segmentation. These output granularities are meticulously designed, each serving a specific function in the decision logic. Specifically, the phase and step provide macro‐level contextual priors for decision‐making, while the action triplet represents the atomic unit of ongoing maneuvers, serving as the decision trigger. Complementarily, scene segmentation supplies the essential spatial localization. We report the HGPNet results in three settings. We first evaluate task‐specific performance under single‐task training and evaluation. Generalization is then assessed using cross‐dataset and cross‐procedure benchmarks. Finally, we report joint multi‐task outputs under unified training, demonstrating comprehensive scene understanding in a single model. To support this evaluation, we curated an expert‐annotated hierarchical granularity dataset, termed LN‐F30, as publicly available datasets with comprehensive multi‐level annotations remain scarce. LN‐F30 is organized from the perspective of autonomous assistance requirements, merging anatomically distinct but operationally similar structures into unified categories to reduce unnecessary class ambiguity. Details of the dataset construction and label taxonomy are provided in Section S1. Each task is evaluated using representative metrics that are standard in the surgical scene understanding field. For surgical phase recognition, we report accuracy, precision, recall, and the Jaccard index. For surgical step recognition, we report accuracy. For both phase and step recognition, we additionally report area under the receiver operating characteristic curve (AUROC) performance. For action triplet recognition, we use mean average precision (mAP). For surgical scene segmentation, we report mean intersection over union (mIoU), mean dice coefficient (mDice), and mean boundary F1‐score (mBF1). Formal definitions of all metrics are provided in Section S2.

**FIGURE 2:**
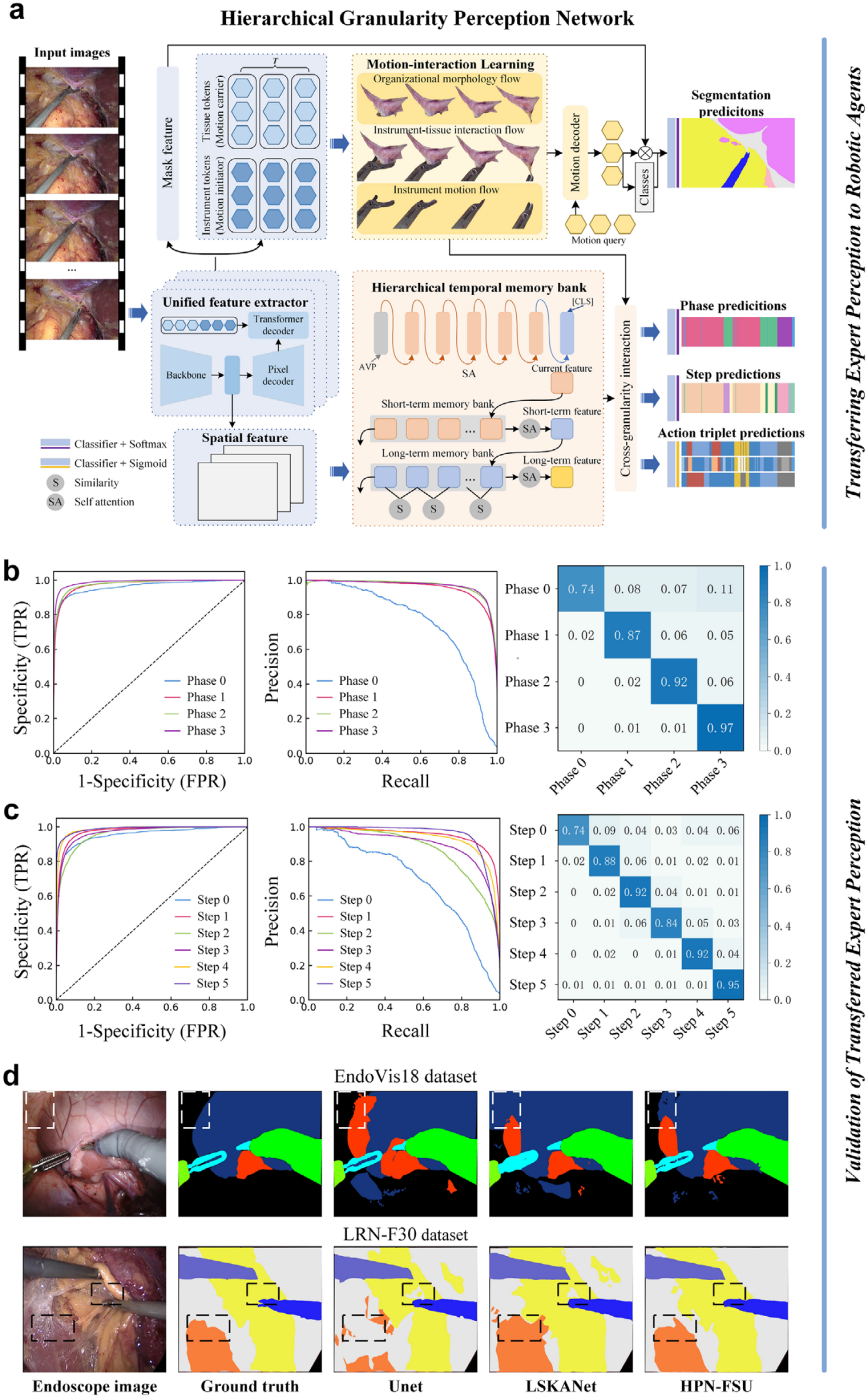
Results of hierarchical granularity surgical scene understanding. (a) The overall framework of the HGPNet. The model employs a shared encoder to simultaneously output predictions across four granularities. It integrates a MIL module to enhance pixel‐level segmentation by capturing bidirectional instrument‐tissue interactions, and a HTMB to provide multi‐scale temporal features for sequence‐level recognition. Furthermore, it leverages cross‐granularity information interaction to facilitate collaborative learning. (b) Quantitative evaluation of the surgical phase recognition task, including the ROC curve, PR curve, and confusion matrix. (c) Quantitative evaluation of the surgical step recognition task, including the ROC curve, PR curve, and confusion matrix. (d) Qualitative visualization of surgical scene segmentation results.

#### 2.1.1 Task‐Specific Performance Under Single‐Task Evaluation

On surgical phase recognition, HGPNet achieves an accuracy of 93.5%, as reported in Table S1. To further assess discriminative performance, we analyze results for each phase. The AUROC values for the four phases are 0.96, 0.97, 0.98, and 0.99, demonstrating strong separability across decision thresholds, as shown in Figure 2b. Precision–recall analysis also indicates robust performance on the key operative phases (Phases 1–3), where high precision is maintained even at high recall. Performance is comparatively lower for the idle phase (Phase 0). This trend is quantitatively supported by the confusion matrix in Figure 2b. Recall remains high for the core operative phases, reaching 87%, 92%, and 97% for Phases 1, 2, and 3, respectively, whereas recall for Phase 0 is 74%. Frame‐level visualization reveals typical failure cases in Figure S2. When the FoV is obscured by blood or surgical smoke, the surgical procedure may temporarily enter an idle phase. Residual visual cues can still cause the model to predict a neighboring active phase. These episodes typically occupy only a brief portion of the overall procedure, resulting in relatively few training samples. This scarcity may limit the model's ability to learn reliable representations for the idle phase. Nevertheless, as the idle phase does not involve critical operative actions, such misclassifications are unlikely to materially affect downstream assistance autonomy.

On surgical step recognition, HGPNet reaches 88.7% accuracy, as reported in Table S2. Since steps provide finer temporal granularity than phases, the task is inherently more challenging, and performance decreases slightly compared to phase recognition, while remaining at a high level. Step‐wise analysis in Figure 2c shows that AUROC exceeds 0.90 for all steps, and AUPR for key steps remains above 0.90, consistent with the strong behavior observed in phase recognition.

On surgical action triplet recognition, HGPNet achieves a component‐association mAP of 81.3%, as reported in Table S3. Triplet recognition is widely regarded as one of the most challenging fine‐grained surgical scene understanding tasks, and top‐performing methods on the CholecTriplet challenge have historically reported substantially lower mAP, typically around the 40% range [36, 37]. This difficulty arises from two primary bottlenecks. First, modeling the structured association among instrument‐verb‐target imposes heavy demands on spatiotemporal reasoning. Second, existing datasets often adopt overly fine‐grained clinical taxonomies in which many categories are visually similar and difficult to separate. HGPNet is strategically designed to dismantle these specific barriers: we leverage pixel‐level motion information to robustly model dynamic component associations, and simultaneously adopt the downstream‐task‐oriented dataset restructuring strategy to minimize semantic ambiguity. Together, these design choices yield a substantial performance improvement and make action triplet recognition more practically usable as a fine‐grained semantic input for surgical robot autonomy.

On surgical scene segmentation, HGPNet achieves 78.1% mIoU, 86.1% mDice, and 81.1% mBF1, as summarized in Table S4. These results indicate accurate region identification and high‐quality boundary delineation, both critical for downstream autonomous view assistance. Qualitative results in Figure 2d show that in regions with active intsrument motion, HGPNet leverages explicit motion cues and instrument‐tissue interaction features to alleviate boundary adhesion issues commonly observed in static models that lack temporal evidence. For static background tissue far from operation region, the model exploits motion‐absent appearance cues as a temporal‐consistency prior, stabilizing large background regions over time.

#### 2.1.2 Task‐Specific Generalization Under Cross‐Dataset Evaluation

To evaluate robustness across data distributions and surgical types, we benchmark HGPNet against SOTA methods on multiple public datasets, as summarized in Tables S1– S4. These datasets span diverse surgical procedures, including Cholec80 [38] and CholecT45 [39] for LC, AutoLaparo [40] for cataract surgery, PSI‐AVA [41] for robotic radical prostatectomy, and EndoVis18 [42] for robotic animal kidney procedures. HGPNet demonstrates strong performance across diverse surgical scene understanding benchmarks. For surgical phase recognition, it achieves 92.8% accuracy on Cholec80 and 85.7% on AutoLaparo, while exhibiting improved stability with a standard deviation of 5.8%. For surgical step recognition on PSI‐AVA, HGPNet reaches 61.9% accuracy. For action triplet recognition on CholecT45, it obtains a mAP of 48.1%. For surgical scene segmentation on EndoVis18, HGPNet attains 68.5% mIoU and a boundary F1‐score of 77.2%. Collectively, these results indicate that HGPNet performs consistently well across tasks and datasets, compares favorably with established baselines, and generalizes well across multiple surgical procedures and scenarios.

We attribute this cross‐procedure generalization in part to the medically grounded design of HGPNet. Specifically, HGPNet models heterogeneous temporal characteristics in surgical workflows while explicitly capturing instrument‐tissue motion interactions. These patterns reflect general clinical priors that underlie the operative logic shared by many surgical procedures, rather than being tied to a single procedure‐specific visual appearance. By embedding such clinical priors into the network design, HGPNet can naturally transfer learned representations and maintain robust performance across diverse surgical scenarios.

#### 2.1.3 Joint‐Task Performance Under Multi‐Task Evaluation

As a unified scene understanding model, HGPNet is trained jointly to output hierarchical granularity surgical scene information. On LN‐F30, joint training yields final performance of 93.9% for phase recognition, 89.4% for step recognition, 84.1% for triplet recognition, and 75.9% mIoU for segmentation, as summarized in Table S5. Relative to single‐task training, joint training improves phase, step, and triplet recognition by 0.4%, 0.7%, and 2.8%, respectively. The largest gain is observed for triplet recognition, consistent with its reliance on short‐term motion cues and its sensitivity to instantaneous motion information provided by segmentation. Although segmentation mIoU decreases slightly under multi‐task weight balancing, this trade‐off is favorable given the consistent improvements across the recognition tasks.

Given the stringent real‐time requirement of the surgical autonomy task, we additionally evaluated the computational efficiency of HGPNet on a workstation equipped with an NVIDIA RTX 4090 GPU. As comprehensive surgical scene understanding models remain scarce, a common alternative is to obtain multi‐granularity outputs by assembling multiple task‐specific models in either serial or parallel execution. We therefore benchmarked HGPNet against two representative multi‐model baselines: a classical lightweight combination of Trans‐SVNet [43] + Rendezvous [39] + U‐Net [44], and a parameter‐intensive SOTA‐style combination of TAPIR [41] + LAM‐BASE [45] + LSKANet [46]. As shown in Tables S5 and S6, HGPNet achieves a favorable performance‐efficiency balance with 165.8M parameters, 26.7 ms inference latency, and 12.8 GB peak GPU memory usage. Its computational profile lies between the classical lightweight baseline and the SOTA‐style baseline, while achieving higher performance on each task. The classical baseline is computationally efficient but underperforms across tasks, whereas the SOTA‐style baseline is accuracy‐competitive yet too resource‐intensive to reliably meet real‐time requirements. Collectively, these results highlight the practical advantage of HGPNet.

### 2.2 Visual‐Attention Behaviors

Upon acquiring comprehensive scene perception, assistance decision‐making necessitates inferring the specific scene information within the surgeon's focus. This inference predicates on a systematic modeling of the surgeon's underlying visual attention behaviors. To this end, we captured intraoperative gaze data via eye tracking, treating fixations as a proxy for visual attention. By aligning these gaze patterns with the hierarchical scene semantics provided by HGPNet, we uncovered implicit correlations between the surgeon's focus and the evolving surgical context. These correlations were distilled into an attention prior to guide autonomous actions, characterizing three distinct behaviors: triplet‐conditioned dynamic attention, phase‐conditioned focal attention, and a personality‐conditioned attention‐to‐action policy that maps attentional targets to view assistance actions.

#### 2.2.1 Eye‐Tracking Acquisition and Analysis Setup

As shown in Figure 3a, the eye‐tracking experimental platform integrates a eye tracker, a commercial laparoscopic imaging system, and a laparoscopic simulator equipped with an organ‐mimicking phantom. We recruited senior attending surgeons to perform standardized simulated laparoscopic procedures while synchronously recording their gaze data. As acquiring high‐quality eye‐tracking data in the operating room is challenging due to personnel interference and the eye tracker working‐distance constraints, we additionally collected gaze patterns from the same surgeons while they viewed real surgical videos. To avoid behavioral bias caused by real‐time gaze feedback, no gaze markers were overlaid on the images during data collection.

**FIGURE 3:**
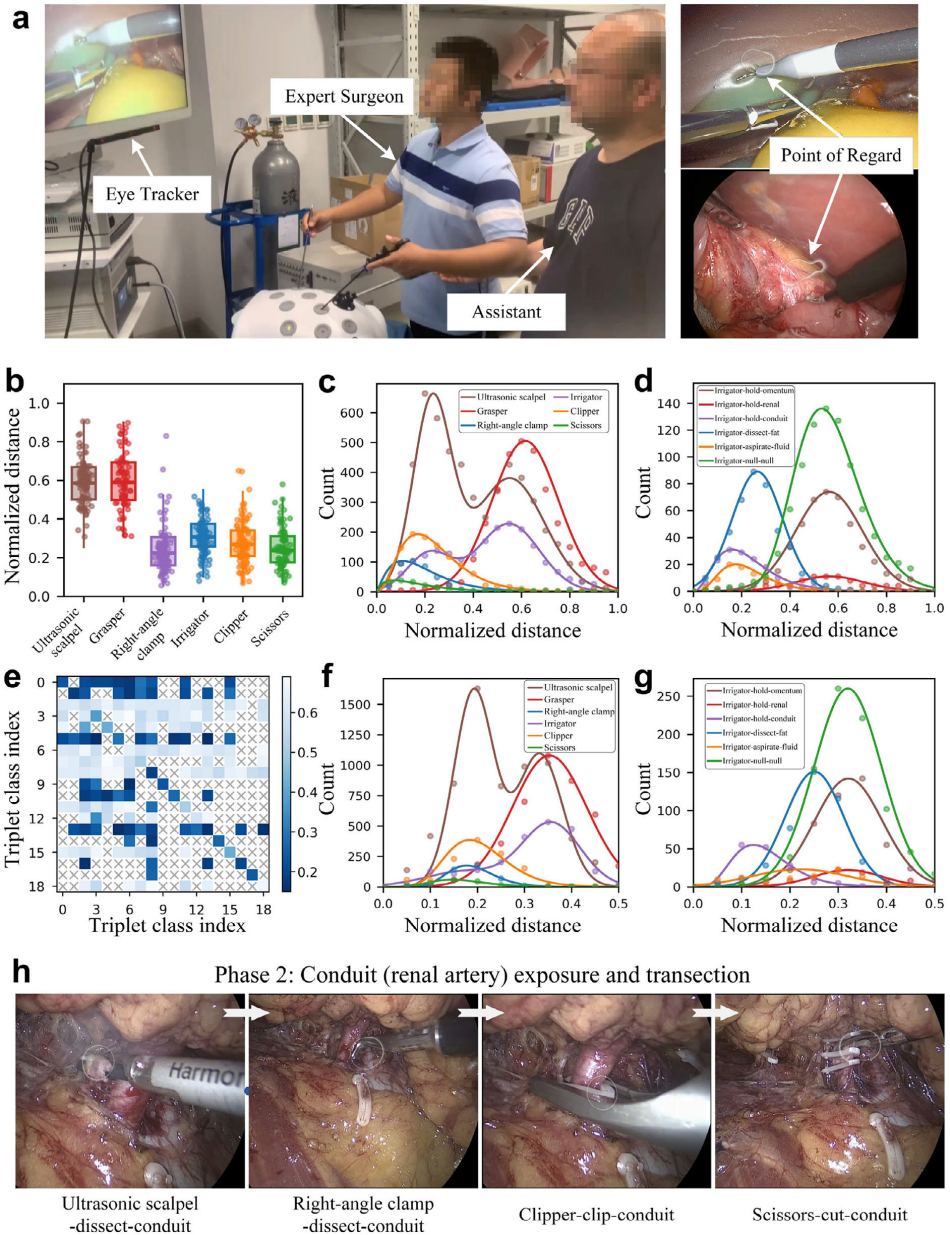
Systematic analysis of surgeon intraoperative visual attention behaviors. (a) The eye‐tracking experimental platform setup. (b) Box plots illustrating the distribution of the normalized distance between the operating region (OR) and the point of regard (POR) across different instrument types. (c) Frequency distribution of the OR‐to‐POR distance across different instrument types. (d) Frequency distribution of the OR‐to‐POR distance across different action triplets involving the irrigator. (e) Pairwise statistical comparison matrix of OR‐to‐POR distances among different action triplets. (f) Frequency distribution of the OR‐to‐center distance across different instrument types. (g) Frequency distribution of the OR‐to‐center distance across different action triplets involving the irrigator. (h) Representative visualization of POR within the conduit exposure and transection phase. The statistical variance observed in (b) (e) demonstrates that visual attention is strictly triplet‐conditioned. The qualitative shifts in (h) highlight phase‐conditioned dynamics. The distributional disparity between the surgeon's gaze focus in (c)/(d) and the resulting view composition in (f)/(g) indicates that the attention‐to‐action policy is not a direct mapping, but rather a dynamically modulated personality‐conditioned policy derived from the surgical context.

To extract common visual‐attention behaviors that generalize across procedures, we conducted experiments on both LN and LC. In the simulation environment, frames from the simulated procedures were manually annotated following the same criteria as LN‐F30. For real surgical videos, LN cases were sampled from the LN‐F30 dataset, whereas LC cases were sampled from the Cholec80 dataset family. After temporal synchronization and spatial registration between gaze data and the endoscopic images, we preprocessed the raw eye‐tracking signals using noise removal, smoothing, and invalid‐segment filtering. This resulted in 938 annotated frames from simulated procedures and 6,471 frames from video viewing for subsequent statistical analysis.

#### 2.2.2 Behavior 1: Triplet‐Conditioned Dynamic Attention Behavior

Based on the eye‐tracking analysis, we found that surgeons' intraoperative visual attention is organized around surgical action triplets. Visual attention changes systematically with variations in any instrument‐verb‐target component. Moreover, as laparoscopic procedures typically involve bimanual operation, co‐occurring triplets further modulate visual attention, leading to context‐dependent shifts in fixation allocation. Notably, a subset of triplets consistently attracts visual attention regardless of their co‐occurrence context, indicating context‐invariant attentional saliency.

To quantify the contribution of each triplet component, we measured the distance between the eye‐tracked point of regard (POR) and the instrument's operating region (OR), approximated by the tool tip. Smaller distances indicate stronger attentional allocation to the ongoing manipulation area. As shown in Figure 3b, instruments such as right‐angle forceps, clipper, and scissors exhibited relatively small distances (0.19−0.27), whereas grasper showed the largest distance (0.64±0.14), with the ultrasonic scalpel and the irrigator lying in between (0.43−0.49). The frequency distributions of the OR‐to‐POR distance (Figure 3c) further reveal deeper patterns of intraoperative visual attention. The ultrasonic scalpel exhibits a characteristic bimodal distribution: a first peak around 0.23 (47.4%, σ=0.08), corresponding to a high‐attention state during critical tissue dissection, and a second peak around 0.55 (52.6%, σ=0.14), corresponding to a low‐attention state when the instrument is idle. Similarly, the irrigator also shows a bimodal pattern, but with markedly imbalanced mode proportions. The dominant and secondary peaks are located around 0.55 (71.5%, σ=0.13) and 0.22 (28.5%, σ=0.09), respectively, aligning with different operative states such as exposure assistance vs. fluid aspiration. These distributions show that the same instrument can be associated with distinct attention levels depending on the ongoing action. Moreover, even under the same instrument‐verb pairing, the target tissue substantially altered attention allocation. As shown in Figure 3d, triplet irrigator‐hold‐conduit yielded shorter distances (0.24±0.12) than irrigator‐hold‐omentum (0.58±0.14), corresponding to an approximately 2.4‐fold difference. Together, these results show that intraoperative visual attention depends on the full triplet configuration rather than any single component alone.

As surgeons operate bimanually, triplets typically co‐occur in pairs. We therefore quantified visual attention under triplet co‐occurrence by computing the conditional fixation distance $d_{i|j}$ for each triplet pair and visualizing the results as a pairwise matrix (Figure 3e). Here, $d_{i|j}$ denotes the mean distance between the POR and the region associated with triplet i, conditioned on the co‐occurrence of triplets i and j. The matrix shows that fixation allocation varies with the concurrent triplet configuration. For example, triplet 9 grasper‐grasp‐kidney exhibits a shorter conditional distance when co‐occurring with triplet 6 ultrasonic scalpel‐null‐null (0.37±0.19), whereas the conditional distance increases to 0.67±0.12 when the ultrasonic scalpel is actively engaged in dissection (triplet 0 ultrasonic scalpel‐dissect‐omentum). Similar context‐dependent shifts appear across multiple triplet pairs. In addition, the matrix reveals a subset of triplets with consistently small fixation distances across co‐occurrence contexts, indicating context‐invariant attentional attraction. For example, irrigator‐aspirate‐fluid (0.25±0.03) and clipper‐clip‐conduit (0.22±0.03) maintain stable, close fixation distances with low variability (standard deviation <0.05), serving as persistent attentional anchors.

Based on the above results, we identify the first visual‐attention behavior, termed triplet‐conditioned dynamic attention behavior. This behavior arises from the inherently bimanual nature of surgical execution, where each instrument‐verb‐target triplet represents the action semantics of a single‐hand operation and thus constitutes a primary unit of operative intent. Accordingly, this attention behavior can be naturally characterized by the triplet distribution predicted by HGPNet.

#### 2.2.3 Behavior 2: Phase‐Conditioned Focal Attention Behavior

Our results further indicate that the attention‐capturing effect of several high‐saliency triplets is phase‐dependent. During the conduit (including the renal artery, renal vein, and ureter) exposure and transection phase, these critical tubular structures act as spatial anchors of visual attention. As illustrated in Figure 3h, although the active triplets switch across a sequence of core actions, the POR remains consistently concentrated around the same vascular vicinity.

Quantitatively, within this phase, 73.3%±5.2% of point of regard (POR) fall within a vessel distance of less than 0.3, and 51.7%±10.9% fall within less than 0.1, indicating sustained focal attention on the vascular region. The remaining gaze points are primarily observed during tool exchanges or brief tracking drifts, rather than reflecting a systematic shift of attention away from the vascular hotspot. Together, these results show that, within specific procedural windows, surgeons stabilize visual attention on a phase‐specific anatomical region of interest, despite rapid switching among individual triplets. Overall, these results demonstrate a phase‐conditioned focal attention pattern, where gaze remains anchored to key tubular anatomy throughout the exposure‐transection window despite triplet switching.

#### 2.2.4 Behavior 3: Personality‐Conditioned Attention‐to‐Action Policy

Behavior 1 and 2 characterize how surgeons allocate visual attention, we further quantified how this visual attention is mapped to view assistance actions. Specifically, we analyzed the distance from each instrument operating region to the image center (Figure 3f) and compared it against the corresponding POR distance distributions relative to the same operating region. As shown in Figure 3c,f, the two distributions are largely consistent. This indicates that operating regions receiving visual attention tend to trigger view assistance actions that recenter these regions. For example, attention attracting instruments such as clipper and scissors tend to exhibit smaller distances to the image center, whereas auxiliary instruments such as graspers show larger center distances, consistent with their weaker POR coupling.

However, the results also show pronounced deviations from this trend, most prominently for the irrigator. Although its OR‐to‐POR distance shows a bimodal pattern (Figure 3c), its OR‐to‐center distance remains largely in the farther range (0.36±0.12, Figure 3f). This mismatch indicates that, in a non‐negligible subset of frames, the attended region is not physically re‐centered by view assistance actions. During diffuse, multi‐point bleeding management with the irrigator, surgeons often maintain a stable global view while addressing bleeding sites sequentially. In this setting, the view is not repeatedly re‐centered on each local suction action, which helps preserve situational awareness of the overall bleeding field. Similarly, during blunt dissection with the irrigator (see Figure S3), the instrument often undergoes large excursions and can move toward the periphery of the FoV. As this manipulation is typically transient, the surgeon's visual attention returns to the central operative region immediately afterward, and a view assistance action is often unnecessary.

Overall, these results suggest that visual attention and view assistance actions are not linked by a fixed one‐to‐one mapping. Instead, view assistance is dynamically configured according to the current surgical context. In core high‐risk operations, view assistance follows a strict centering regime, closely coupling adjustments to the attentional focus to keep critical targets near the image center. In tasks requiring multi‐region coordination or global monitoring, view assistance follows a gentle centering regime, tolerating a sustained attention offset to maintain global stability. We summarize this context‐dependent regime switching as a personality‐conditioned attention‐to‐action policy.

### 2.3 In Vivo Animal Trials

Despite extensive progress in autonomous laparoscopic view management, demonstrating fully autonomous view assistance throughout an entire soft‐tissue procedure in vivo setting remains rare. We therefore conducted in vivo porcine trials of LN to evaluate intent‐to‐action transfer, the final stage of our expertise transfer framework for autonomous surgical assistance. The study protocol was reviewed and approved by the ethics committee of the Affiliated Hospital of Qingdao University. All procedures were performed by surgeons with laparoscopic experience under full life‐support and monitoring, including continuous ECG, blood pressure, and oxygen saturation monitoring, together with mechanical ventilation and anesthesia management by a dedicated anesthesia team.

The experimental setup is shown in Figure 4a. Intra‐abdominal images were acquired using an Olympus VISERA 4K UHD endoscopic imaging system. The laparoscope was mounted on a JAKA ZU 3 collaborative robot with six degrees of freedom (DoF) via a custom 3D‐printed adapter. The endoscopic video stream served as the sole perception input to the expertise transfer framework, which generated real‐time view assistance commands to the robot. After establishing pneumoperitoneum and placing trocar ports with the animal in the lateral decubitus position, the primary surgeon localized the kidney and completed the nephrectomy following standard clinical practice. In the experimental group, view assistance was controlled by the proposed system. In the control group, view assistance was performed by a dedicated human assistant. To assess robustness across surgeon experience levels and animal conditions, the experimental group comprised two trials (group I and group II). Group I was performed by a senior expert surgeon on a 42 kg male pig, whereas Group II was performed by a surgeon with standard clinical experience on a 38 kg female pig. To reduce inter‐animal variability, the control trial was performed on the contralateral kidney of the same animal used in group II.

**FIGURE 4:**
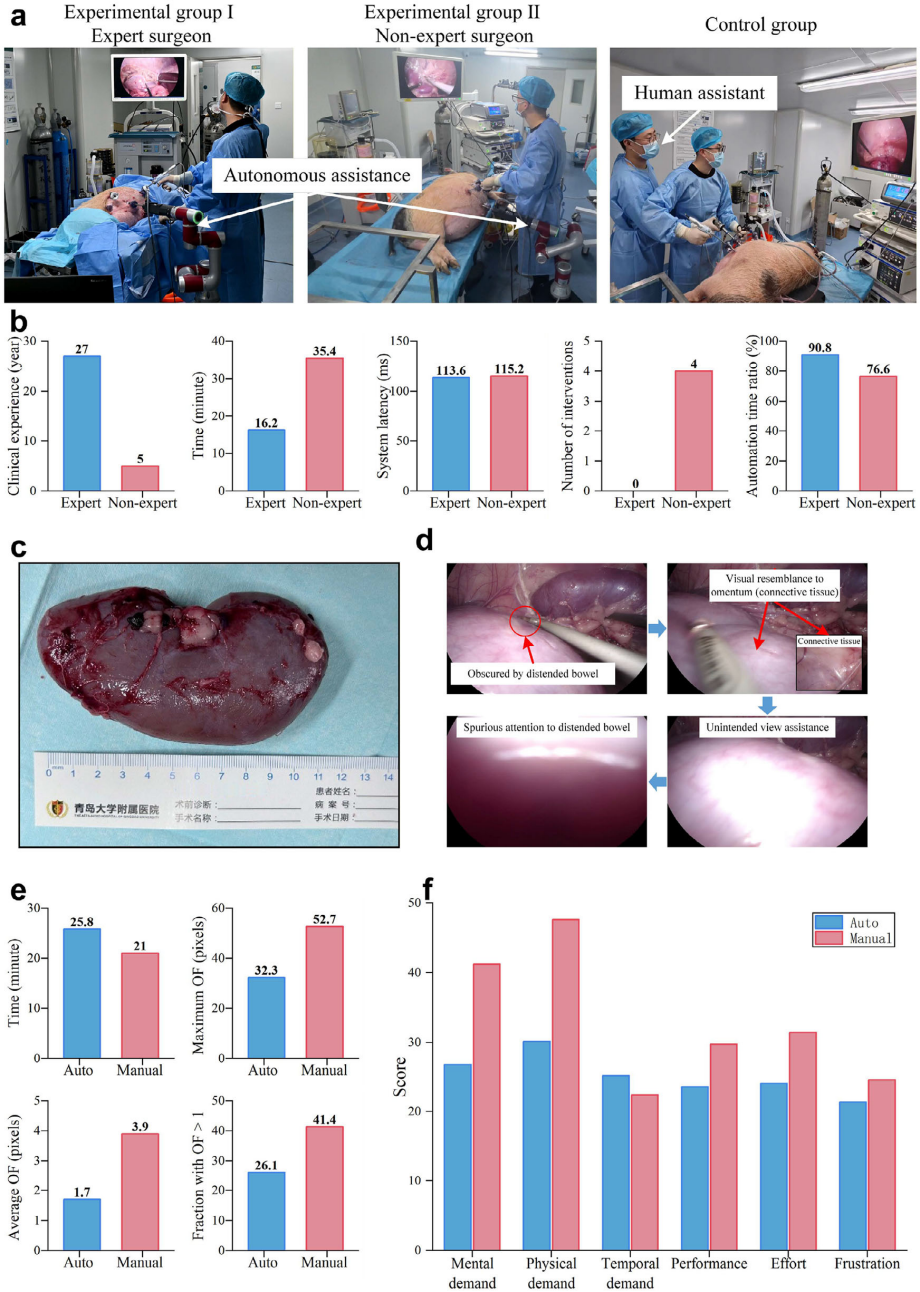
Overview of in vivo validation results. Our framework delivered autonomous view assistance throughout the entire laparoscopic nephrectomy. (a) Experimental setup. Our framework provided autonomous assistance to both an expert surgeon (group I) and a non‐expert surgeon (group II), while the control group relied on a human assistant. (b) Adaptability assessment across surgeon experience levels. All procedures were successfully completed with high automation time ratios. (c) The intact kidney successfully resected in experimental group I. (d) Analysis of a failure case in the non‐expert group. The anomalous event of bowel distension caused visual ambiguity, triggering unintended view assistance. (e) Quantitative evaluation of visual stability. The proposed method yields significantly lower optical flow metrics compared to manual control, indicating superior visual stability. (f) NASA‐TLX workload assessment. Comparative results highlight that our framework reduces the surgeon's mental and physical demand compared to human assistance.

#### 2.3.1 Autonomous Assistance Throughout the Laparoscopic Nephrectomy Procedure

Across all autonomous trials conducted with surgeons at different experience levels, surgeons successfully completed full LN procedures with view assistance provided by the proposed framework. The resected kidneys were structurally intact with a clearly preserved capsule; an example specimen from the expert surgeon group is shown in Figure 4c. The assistant achieved real‐time responsiveness, with end‐to‐end response times consistently below 120ms. Throughout the procedures, the autonomous view assistance actions preserved the procedural flow and did not physically interfere with the surgeon manipulation. Autonomous view assistance accounted for the majority of operative time. As shown in Figure 4b, the intervention free automation ratio reached 90.8% in the expert surgeon trial and 76.6% in the non‐expert surgeon trial. No tissue injury attributable to autonomous view assistance actions was observed, and both animals survived intraoperatively.

We next present representative in vivo segments to illustrate how our framework configured assistance actions under different surgical contexts. A representative single view assistance event is shown in Figure 5a. In this scene, the framework recognized two co‐occurring surgical action triplets with a clear primary‐secondary relationship: an auxiliary operation grasper‐grasp‐omentum, and a key operation ultrasonic scalpel‐dissect‐omentum. When the key operative interaction region moved toward the FoV periphery, our framework triggered a view assistance action and re‐centered the FoV onto the ultrasonic‐scalpel working region, rather than focusing on the auxiliary grasper region. This strict re‐centering configuration is desirable during critical dissection because it prioritizes visibility of the dominant operative interaction. A continuous segment composed of multiple such assistance events is shown in Figure 5b. This excerpt covers a 146 s interval of uninterrupted bimanual manipulation in a typical critical dissection stage. Despite substantial changes in operative regions and viewing geometry, our framework consistently prioritized the key interaction and suppressed unnecessary tracking of auxiliary operative regions. As shown in Figure 5b, the resulting FoV assistance trajectory closely overlapped with the surgeon operative trajectory along the kidney contour. These observations exemplify action‐triplet‐conditioned prioritization, expressed through a strict configuration of the personality‐conditioned attention‐to‐action policy.

**FIGURE 5:**
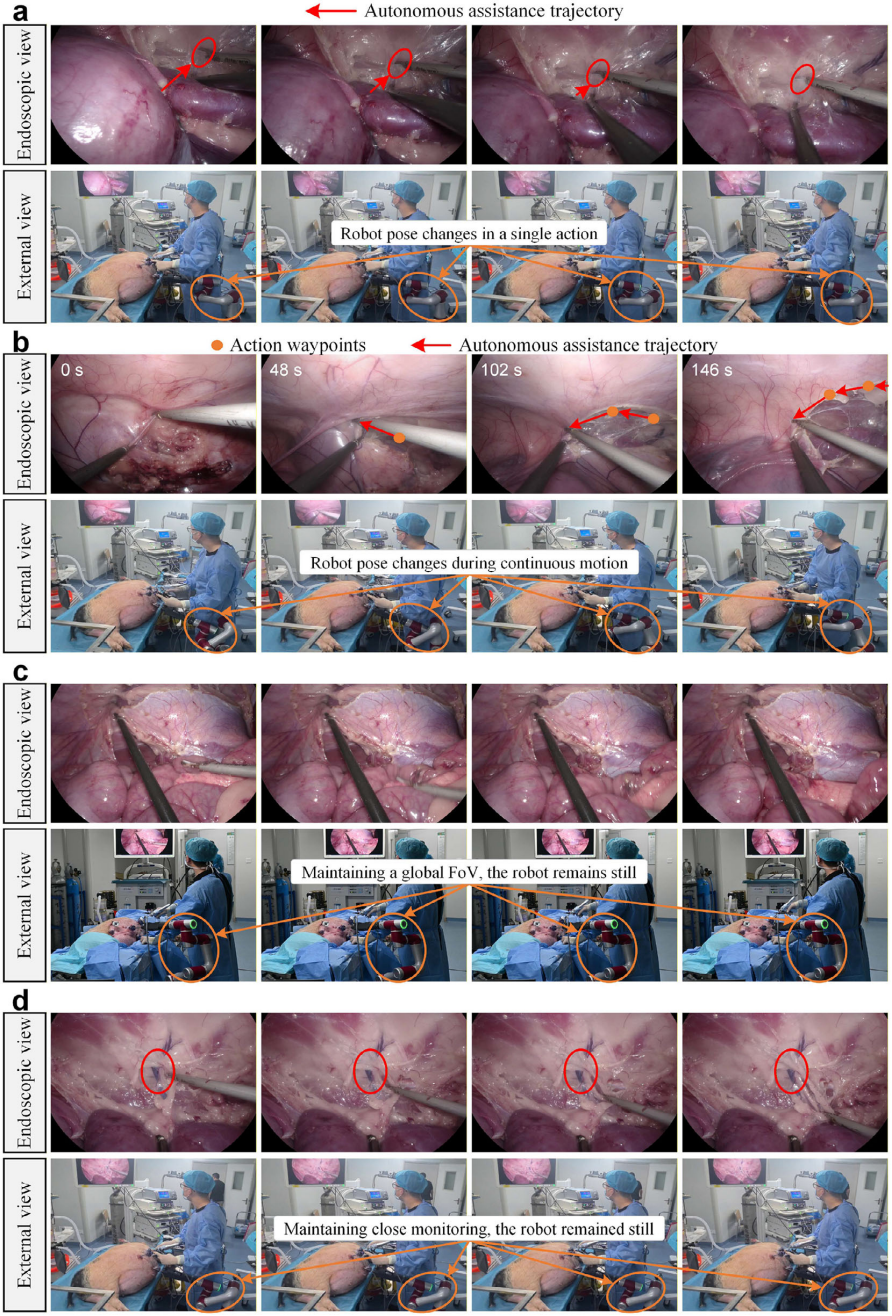
Qualitative analysis of autonomous assistance behaviors generated by our framework. (a) A triplet‐conditioned single assistance action. Our framework activates a strict configuration to intensify focus on the core surgical region. (b) Triplet‐conditioned continuous assistance actions. During the perirenal tissue dissection phase, the generated autonomous assistance trajectory highly aligns with the surgeon's manipulation path along the renal contour. (c) A global‐view maintenance scenario under a gentle configuration. This context requires a broad perspective to retract tissue obscuring the kidney; our framework maintains global stability even when instruments transiently exit the view. (d) A phase‐conditioned anchoring scenario. During the critical conduit exposure and transection phase, our framework persistently anchors its focus on the vital conduit structures.

A contrasting scenario dominated by auxiliary maneuvers is shown in Figure 5c, illustrating global view maintenance cases. When the primary operative region is occluded by surrounding tissues (e.g., the bowel), exposure must be restored. This is typically achieved through coordinated manipulation, where the grasper and ultrasonic scalpel retract or stabilize the occluding tissue. In this setting, the ultrasonic scalpel may move toward the FoV boundary or briefly leave the frame, and the grasper is not necessarily maintained in a centered location. Our framework ignored these short‐lived excursions and maintained a globally informative FoV rather than repeatedly re‐centering on each transient motion. This gentle configuration is clinically meaningful because global stability helps preserve situational awareness while the workspace is being prepared for the next key operative action. These observations likewise exemplify action‐triplet‐conditioned prioritization, expressed through a gentle configuration of the personality‐conditioned attention‐to‐action policy.

A phase‐dependent anchoring scenario is shown in Figure 5d. This segment occurs during the conduit exposure and transection phase, in which a sequence of core actions is organized around a key tubular structure. Although the interaction region of the key triplet ultrasonic scalpel‐dissect‐omentum temporarily moved toward the FoV periphery, the proposed framework prioritized continuous observation of the phase‐relevant anatomical target over momentary tracking of the instrument instantaneous position. By maintaining stable focus on the conduit region, our method kept the most task‐relevant anatomical information near the FoV center across this phase window. This behavior is clinically aligned with the longer‐horizon nature of phase goals, where sustained visibility of a key structure can be more informative than reacting to short‐lived tool excursions. These observations are consistent with phase‐conditioned focal attention behavior.

#### 2.3.2 Robustness Across Surgeon Experience Levels

To evaluate whether the proposed framework can be used by surgeons with different expertise levels, we conducted in vivo LN trials under autonomous view assistance with an expert surgeon and a non‐expert surgeon. The expert surgeon had 27 years of clinical experience and served as the department chair of urology, whereas the non‐expert surgeon had 5 years of experience.

In the expert surgeon group, the surgeon completed the full LN in 16.2 min after routine setup. View assistance was carried out autonomously throughout the procedure without manual view assistance intervention, achieving an intervention‐free automation ratio of 90.8% (Figure 4b). The human involvement was brief lens wiping when endoscope blur occurred. This high degree of hands‐free autonomy is consistent with the expert's standardized workflow and minimal unnecessary maneuvers, resulting in operative patterns that align well with the assistant's learned priors and decision logic.

In the non‐expert surgeon group, overall collaboration with our framework remained effective and the procedure was completed successfully. Owing to reduced familiarity with the operative workflow, the operative time increased to 35.4 min (Figure 4b). The trial required four manual view‐assistance interventions; consequently, the automation time ratio decreased to 76.6%. These interventions were primarily associated with unexpected intraoperative conditions and subsequent recovery actions. In group II, non‐standard events such as bowel distension and fluid leakage were observed, which were partly attributable to the less experienced surgeon's handling during the procedure. As shown in 4d, the distended bowel exhibited a visual appearance similar to connective tissue, which is merged and annotated as omentum in LN‐F30 dataset. When the surgeon used the ultrasonic scalpel to stabilize the distended tissue, the system misclassified the action as the triplet ultrasonic scalpel‐dissect‐omentum. These persistent misclassifications exceeded the short‐term tolerance safety mechanism, thereby triggering an inappropriate view adjustment that occasionally contacted the distended bowel, followed by lens contamination and image blur. Manual intervention was then performed to clean the lens and restore an appropriate view, after which the procedure proceeded normally. As the laparoscope remained at a safe distance from critical structures; the occasional contact observed here was associated with unusually severe distension. No tissue injury attributable to autonomous view assistance actions was observed. Overall, despite occasional manual interventions, particularly in the non‐expert trial, the proposed framework enabled successful completion of full LN while maintaining autonomous view assistance. These results support its adaptability across surgeons with different experience levels.

#### 2.3.3 Comparison With Human Assistant

In a pilot in vivo comparison, we benchmarked the proposed method against a dedicated human assistant performing manual view assistance. Although exploratory in scale, to the best of our knowledge this is the first paired in vivo comparison of autonomous view assistance vs. human assistant conducted on the bilateral kidneys of the same animal. As in vivo studies of autonomous assistance remain scarce, most prior work has focused on simplified, segment‐level tasks, making it difficult to quantitatively compare performance against human assistance at the procedure level. Consequently, commonly used evaluation metrics have been largely limited to task completion time and subjective workload quantified by NASA‐TLX [47]. Following this convention, we also report these two metrics. In addition, to provide a procedure‐level evaluation from a holistic workflow perspective, we introduce two objective metrics: an optical flow based measure of visual stability and a visual content quality metric based on the spatial distribution of instrument working regions.

As shown in Figure 4e, human assistant yielded a shorter procedure time than the autonomous assistance, with 21.0 min vs. 25.8 min, respectively. The modest increase in time under autonomous assistance was primarily associated with lens wiping events, which were more cumbersome to execute within the autonomous workflow than when performed opportunistically by a human assistant. Despite the longer completion time, our framework achieved better subjective ratings across most NASA‐TLX dimensions (Figure 4f). In particular, self‐reported scores across the mental demand, physical demand, effort, and frustration dimensions were lower. Together, these pilot observations suggest that the proposed method can maintain a clinically effective operative field.

Visual stability is critical for effective surgical assistance, yet objective quantitative measures are still lacking. We therefore propose an optical flow based metric to quantify visual stability. Optical flow estimates per‐pixel displacement between consecutive frames and therefore provides a direct description of inter‐frame motion: when the FoV is unstable, nominally static regions of the scene exhibit abnormal motion signals across the image. To visualize visual stability under comparable surgical context, we analyzed one representative continuous 80‐frame segment per condition (manual vs. autonomous assistance), selected from similar perirenal dissection periods. Figure 6a,b presents endoscopic frames sampled every 20 frames together with the corresponding optical flow maps, where pixel intensity encodes flow magnitude and pixel hue encodes motion direction. In the manual‐assistance condition (Figure 6a), pronounced optical flow signals were observed in regions expected to remain static, including the image periphery (highlighted in Figure 6a). This indicates global view oscillation likely caused by hand tremor of the human assistant, resulting in reduced visual stability. By contrast, under our method (Figure 6b), optical flow activity was largely concentrated around the active ultrasonic instrument, while abnormal motion in nominally static background regions was substantially reduced. Quantitative summaries for these 80‐frame segments are reported in Figure 4e. The manual segment exhibited a mean optical flow magnitude of 3.87 pixels per frame and a 41.39% fraction of pixels with flow magnitude > 1, whereas our methods showed 1.72 pixels per frame and 26.11%, corresponding to reductions of 55.6% and 36.9%, respectively.

**FIGURE 6:**
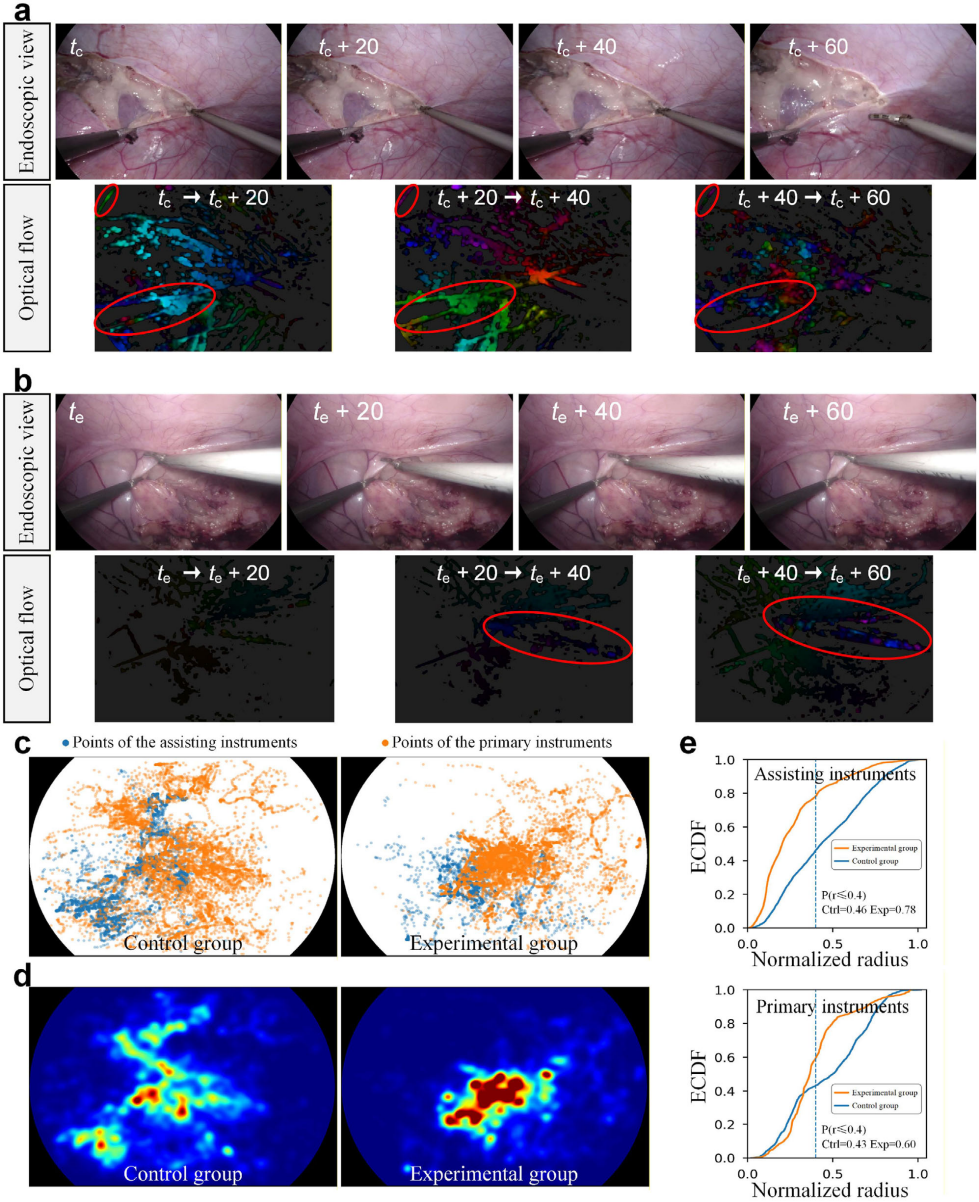
Comparative analysis of visual stability and visual content quality between our framework (experimental group) and human assistance (control group). (a,b) Visual stability assessment using optical flow during similar kidney dissection maneuvers. (a) In the control group, substantial optical flow (high‐velocity motion artifacts) is observed even in static background tissues and the inactive grasper (red contours), attributable to the physiological tremors and fatigue inherent to human assistance. (b) In contrast, the experimental group exhibits optical flow strictly confined to the active ultrasonic scalpel region (red contour), with negligible motion in the background, demonstrating superior visual stability. (c) Scatter plots of instrument operation points. (d) Density heatmaps of instrument operation points. The control group displays a dispersed spatial pattern, whereas the experimental group shows a highly condensed distribution clustered around the visual center, indicating that the autonomous assistance consistently maintains an optimal workspace. (e) Empirical cumulative distribution functions (ECDF) of the normalized radial distance from the image center. The experimental group demonstrates a significantly higher probability mass within the central region compared to the control group.

Visual content quality is also critical, as it directly determines what the surgeon sees over time. We operationalized content quality through the spatial distribution of instrument working regions within the FoV, under the premise that effective view assistance should keep the core operation region compact and preferentially centered. Unlike the illustrative 80‐frame stability snapshot, this content‐quality analysis was computed over the entire perirenal tissue dissection. Instrument working regions were localized at frame level using our HGPNet, and all metrics were computed deterministically from the resulting spatial distributions and applied identically to both conditions. Figure 6c shows frame‐wise spatial samples of instrument working regions throughout the phase. Under human assistance, the working regions exhibit a broader spatial spread with frequent excursions toward the periphery, suggesting that the operative region was less consistently maintained near the image center. In contrast, under our autonomous assistance, the instrument working regions form a more compact distribution with higher density near the center. This observation is further supported by the density maps in Figure 6e. Our autonomous assistance condition shows a pronounced central hotspot, whereas the human assistance condition presents dispersed hotspots spanning a larger fraction of the FoV. To quantify centrality, we computed the empirical cumulative distribution function (ECDF) of the normalized radial distance r from the image center, where smaller r indicates more central framing (Figure 6d). Under autonomous assistance, the ECDF curves of instrument working regions are consistently left‐shifted relative to those under manual control, indicating smaller radii across frames. Using a centrality threshold of r≤0.4, the probability mass within the central region increased from 0.46 to 0.78 for the ultrasonic scalpel working region and from 0.43 to 0.60 for the grasper. Beyond frame‐level centrality, we quantified the concentration of instrument activity on aggregated density maps (Table S7). Compared with human assistance, our autonomous assistance yielded a significantly more concentrated spatial distribution. Specifically, our method achieved lower normalized entropy and effective area, alongside a higher Herfindahl‐Hirschman index (HHI), collectively indicating a shift from scattered operation to a sharper, more dominant focus. Notably, the minimal area required to contain 90% of the density mass dropped substantially from 38.5% to 23.1%. This confirms that our framework enables surgeons to maintain a tighter operating envelope around the FoV center, delivering superior view context quality.

Collectively, these in vivo experiments validate the feasibility of the proposed expertise transfer framework for autonomous surgical assistance. Throughout the trials, our framework sustained a high level of autonomy while demonstrating superior view quality compared to a dedicated human assistant.

### 2.4 Phantom‐Based Simulation Experiments

#### 2.4.1 Cross‐Procedure Generalization to Laparoscopic Cholecystectomy

To probe cross‐procedure generalization beyond LN, we further evaluated the proposed framework in a simulated LC workflow. Although LC is generally less technically demanding than LN, it is one of the most commonly performed laparoscopic procedures worldwide and therefore provides a representative and clinically relevant setting for assessing cross‐procedure transfer. We adopted our previously reported LC simulation platform and task design [48] as a standardized testbed, and used it here to evaluate the proposed framework under a distinct workflow. The workflow is illustrated in Figure S4a and comprised liver retraction (three‐position lifting for gallbladder exposure), gallbladder bed dissection (concurrent retraction with hook‐based dissection), and cystic duct isolation simulation.

Furthermore, to benchmark the proficiency level of our autonomous system against human capabilities, we established a comprehensive comparative baseline. To eliminate confounding variables associated with surgical technique, the identity of the primary surgeon remained fixed throughout the evaluation. The surgeon collaborated with our autonomous framework alongside three distinct tiers of human assistants: an expert baseline, comprising a clinical professional with a well‐established history of collaboration with the primary surgeon; a general baseline, represented by a robotics researcher equipped with prior simulation training and collaborative practice; and a novice baseline, consisting of a researcher possessing only a fundamental understanding of the surgical workflow and minimal simulation experience. Each experimental condition consisted of five full procedural episodes. System performance was rigorously quantified using task completion time, average optical flow, number of manual interventions, and peripheral occupancy ratio. Specifically, the peripheral occupancy ratio is defined as the fraction of operative frames in which the core instrument working regions lie within the outermost 15% margin of the image.

As illustrated in Figure 7c, benchmarking our autonomous system against these varying human baselines positioned its operational capability between general proficiency and expert‐level assistance. Regarding procedural efficiency, the autonomous system completed the workflow in 121.6 ± 33.1 s, notably outperforming the novice and general baselines. It executed slightly slower than the expert baseline's 110.2 ± 28.9 s. This was primarily due to the human expert's highly anticipatory skills, which enable them to preemptively adjust the FoV to the next surgical region as the current maneuver nears completion. Nevertheless, our framework demonstrated a clear advantage in visual stability. It recorded an average optical flow of 2.1 ± 0.6 pixels, surpassing human assistants across all levels by effectively minimizing physiological tremors. This consistent stability directly translated to enhanced system performance, necessitating an average of less than one manual intervention per trial. Furthermore, regarding visual framing quality, our algorithm effectively kept critical surgical maneuvers centered, maintaining a notably low peripheral occupancy ratio of 0.06 ± 0.02. This predictive framing capability significantly outperformed the general assistant's ratio of 0.15 ± 0.05 and performed comparably to the high standard of 0.06 ± 0.04 set by the expert baseline. Collectively, these results indicate that our framework not only demonstrates robust cross‐procedure generalization capabilities but also provides near‐expert surgical assistance.

**FIGURE 7:**
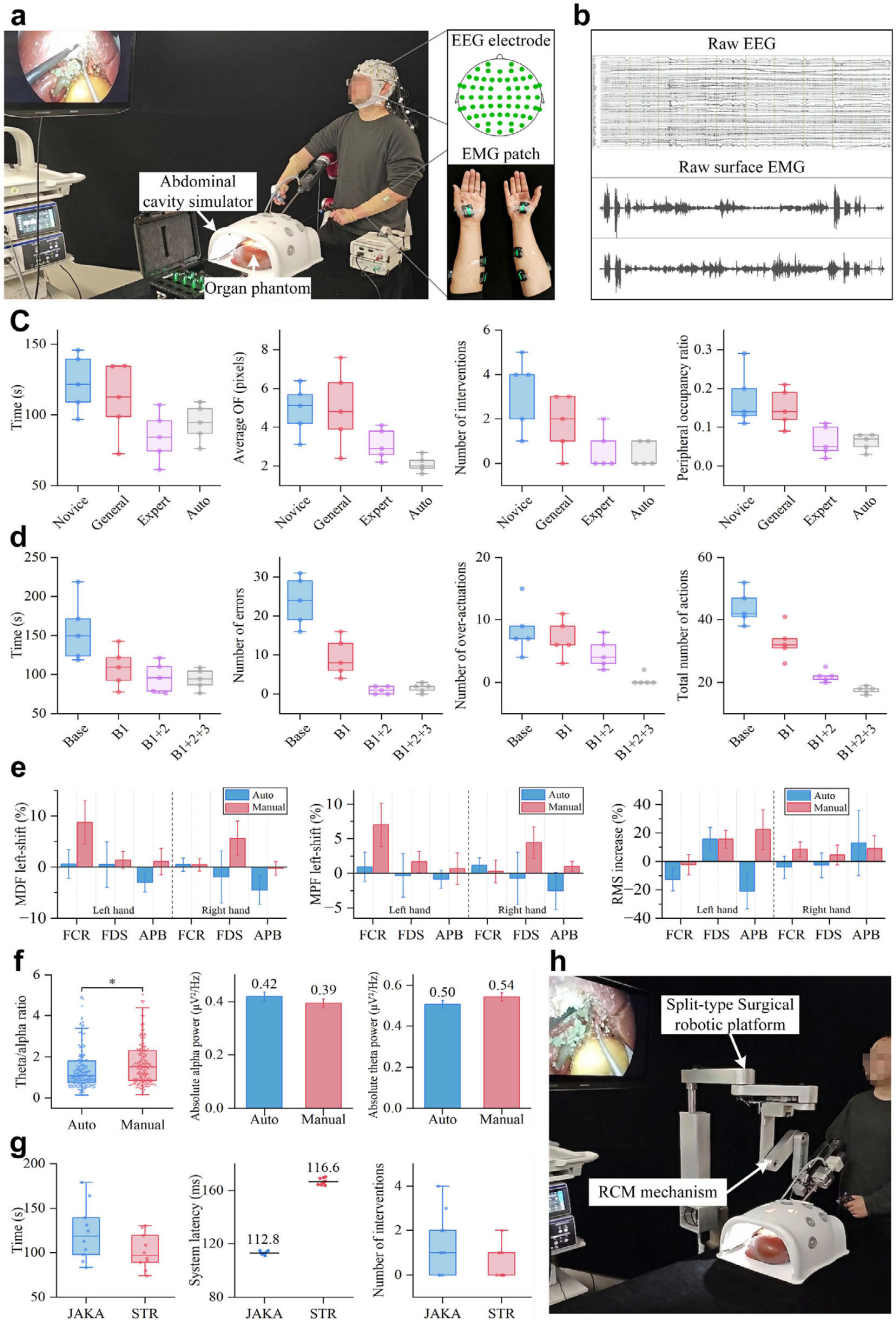
Overview of the phantom‐based simulation experiment results. (a) Experimental setup of the simulation platform. (b) Representative raw traces of the intraoperative EEG and surface EMG signals. (c) Quantitative comparison between the proposed autonomous framework and multi‐level human assistance. The overarching metrics indicate that the autonomous assistance performance positions it between a general and an expert human assistant. (d) Results of the step‐wise ablation study, validating that the progressive integration of the three visual‐attention behaviors substantially improves the overall assistance quality. (e,f) Quantitative assessment of physical fatigue and cognitive load derived from (e) EMG and (f) EEG data, demonstrating that the autonomous method induces a significantly lower physiological and mental burden on the surgeon compared to manual assistance. (g) Quantitative results of the cross‐platform generalization experiments. (h) Experimental setup illustrating the migration of the proposed autonomous framework onto a proprietary split‐type surgical robotic platform.

The quantification of physiological and neural metrics has emerged as a robust paradigm for evaluating intraoperative cognitive burden and systemic surgical workload [49, 50, 51]. Aligning with this approach, to objectively evaluate the systemic ergonomic benefits of the autonomous framework, we quantified the primary surgeon's physical fatigue and cognitive load using surface electromyography (EMG) and electroencephalography (EEG), respectively. The overall experimental setup is illustrated in Figure 7a. Detailed descriptions of the experimental paradigms and signal preprocessing pipelines are provided in Section S6.

For physical fatigue assessment, we continuously monitored critical upper‐limb muscles relevant to surgical manipulation. These specifically included the flexor digitorum superficialis (FDS), the flexor carpi radialis (FCR), and the abductor pollicis brevis (APB). As illustrated in Figure 7e, our autonomous assistance yielded reduced leftward shifts in median frequency (MDF) and mean power frequency (MPF) compared to human assistance, indicating a reduction in induced physical fatigue.Specifically, manual camera control drove the MDF and MPF left‐shifts for the left FCR to 8.7% and 7.1%, respectively. In contrast, the autonomous framework maintained these spectral shifts at lower levels of 0.6% and 0.9%. Time‐domain analysis further corroborated these findings. Whereas human assistance caused a 22.4% increase in root mean square (RMS) amplitude in the left APB, the autonomous system attenuated this muscular tension, achieving a 20.8% reduction. It is worth noting that actual surgical manipulations are dynamic and do not strictly adhere to isolated isometric contractions. Consequently, this inherently introduces abrupt fluctuations in the RMS data.Nevertheless, the overall reduction across these combined physiological metrics demonstrates an ergonomic benefit. The stable visualization provided by our system reduces the need for the primary surgeon to execute additional, compensatory left‐hand movements to maintain an adequate FoV.

Concurrent EEG monitoring provided quantitative neurophysiological evidence regarding cognitive load. The overarching theta/alpha Ratio (TAR) serves as a validated index of mental workload. As shown in Figure 7f, this ratio was significantly lower during autonomously guided operations (p<0.05). Frequency band analysis revealed that the autonomous system reduced absolute frontal theta power (0.50 vs. $0.54\;\mu V^{2}/Hz$), suggesting a decrease in working memory demands and executive control required for spatial compensations. Simultaneously, parietal alpha power was elevated (0.42 vs. $0.39\;\mu V^{2}/Hz$), reflecting a reduction in sensorimotor load and visual fatigue. Collectively, these metrics demonstrate that the visualization provided by our autonomous algorithm effectively mitigates both the physical and cognitive burdens on the primary surgeon compared to conventional manual assistance.

#### 2.4.2 Ablation Study

To evaluate the specific effects of each design variable on overall surgical performance, we conducted a comprehensive ablation study. We established four step‐wise configurations: a frequency‐driven baseline (Base), and configurations progressively integrating our three visual‐attention behaviors (B1, B1+2, and the full model B1+2+3). To eliminate inter‐operator variability, a fixed expert surgeon performed the simulated cholecystectomy workflow across five episodes per configuration. To explicitly capture internal algorithmic accuracy and economy of motion, system performance was evaluated using four targeted metrics: task completion time, total FoV adjustments, number of errors, and number of over‐actuations. Specifically, an error is defined as an erroneous adjustment action triggered by clinically irrelevant regions, whereas an over‐actuation refers to consecutive and redundant micro‐adjustments.

As illustrated in Figure 7d, the step‐wise integration of our visual‐attention behaviors translated into concrete improvements across all systemic metrics. Regarding the number of errors, the frequency‐driven baseline indiscriminately tracked active auxiliary movements, such as tissue grasping for exposure. This strategy resulted in a high error rate of 23.8 ± 5.8. The introduction of behavior 1 substantially decreased this metric to 9.4 ± 4.3. Furthermore, by introducing the surgical phase anchor mechanism in behavior 2, the error rates for the B1+2 and full model configurations exhibited a marked decline to 1.0 ± 0.9 and 1.6 ± 1.0, respectively. This effectively eliminated phase‐incongruent visual noise during critical steps like cystic duct isolation. The evaluation of over‐actuations explicitly highlighted the necessity of the dynamic visual tolerance mechanism introduced in behavior 3. Because procedures like LC typically progress along continuous anatomical contours, the spatial distance between consecutive surgical targets is often extremely short. Relying solely on the B1+2 configuration indiscriminately executed every triggered command, inevitably leading to hyper‐reactive micro‐adjustments with a recorded 4.6 ± 2.2 over‐actuations. The full B1+2+3 configuration effectively circumvented this issue, drastically reducing over‐actuations to 0.4 ± 0.8 and maintaining essential FoV requirements while delivering superior economy of motion.

Critically, these micro‐level algorithmic deficiencies in the baseline method manifested as macroscopic inefficiencies. The over‐fixation on auxiliary movements inadvertently compromised primary site visualization, forcing the surgeon to perform compensatory actions to re‐expose the target anatomy. This triggered a vicious cycle of inefficient camera tracking and surgical workflow disruption. Consequently, both task completion time and total FoV adjustments experienced a severe surge, peaking at 156.4 ± 35.8 s and 44.0 ± 4.9, respectively. This phenomenon elucidates why existing frequency‐based methods rarely succeed in complex in vivo trials. Ultimately, the progressive integration of our clinically derived visual‐attention behaviors systematically disrupted this cycle. We consistently observed a robust, step‐wise decline in both surgical time and adjustment frequency, confirming that our framework fundamentally upgrades actual surgical performance.

#### 2.4.3 Cross‐Platform Generalization

To further validate the cross‐platform robustness of our framework, we conducted additional tests utilizing an in‐house developed split‐type robotic (STR) platform. Unlike the general‐purpose JAKA collaborative robot, this dedicated STR system features a specialized remote center of motion (RCM) mechanism. This architecture provides an expanded operative workspace and significantly reduces spatial intrusion into the primary surgeon's working area. The experimental setup of the STR platform is illustrated in Figure 7h. We replicated the simulated cholecystectomy workflow but explicitly instructed the operators to perform surgical movements with substantially larger amplitudes. This design deliberately stress‐tested the kinematic execution near the boundaries of the robot's workspace. To ensure baseline familiarity with the robotic systems, we recruited an expert surgeon and a core researcher who both participated in the initial simulation experiments. Each operator completed five trials on each platform. System performance was evaluated across three metrics: task completion time, average system latency per action, and the frequency of manual interventions.

As shown in Figure 7g, the comparative validation demonstrated ergonomic and efficiency advantages when utilizing the specialized surgical platform. Under the STR setup, the average task completion time decreased from 122.6 ± 31.8 s to 101.4 ± 18.8 s, exhibiting a notably tighter data distribution. Furthermore, the STR platform effectively accommodated the amplified movements and suppressed spatial conflicts. Specifically, 60% of the trials using the STR setup required zero manual interventions, in stark contrast to the higher frequency of interventions observed under the JAKA platform. While the custom STR prototype recorded a higher average latency 166.8 ± 2.4 ms compared to the highly optimized, commercial‐grade JAKA system 113.2 ± 1.1 ms, this response time remained entirely acceptable for surgical assistance tasks. Collectively, these results verify that our framework is highly hardware‐agnostic at the decision‐making level. In addition, pairing it with dedicated surgical robotic systems further amplifies overall clinical efficiency and ergonomics.

## 3 Discussion

In this work, we propose an expertise transfer paradigm to explore autonomy for procedure‐spanning tasks. Our framework explicitly reconstructs the expertise by hierarchically integrating comprehensive surgical scene understanding, context‐dependent visual attention patterns, and transparent action selection logic. Under this paradigm, we successfully demonstrate procedure‐level autonomous view assistance throughout complete soft‐tissue operations in vivo trials. Compared with human assistance, our method was associated with lower subjective workload and consistently improved view quality.

Unlike episodic sub‐tasks that may rely on localized visual cues, procedure‐spanning autonomy necessitates a significantly more comprehensive and real‐time understanding of the surgical scene. Since the assistance task demands synergy with the lead surgeon, it necessitates not merely the localization of instruments and tissues, but also an acute perception of the dynamic evolution of the surgical workflow and the surgeon's current operative actions. To this end, we developed HGPNet to jointly capture surgical phase, step, action triplets, and scene segmentation. Rather than training these tasks in isolation, we designed task‐differentiated feature processing and cross‐granularity interaction mechanisms to coordinate learning across distinct granularities. However, balancing the incorporation of such rich feature representations with the strict computational efficiency required for clinical deployment remains a long‐standing challenge. We drew inspiration from human memory mechanisms, specifically the selective consolidation and forgetting properties that enable efficient learning under limited neural capacity. Building on these biological principles, we developed a hierarchical temporal memory bank approach. By dynamically reinforcing or filtering historical information based on context, this module achieves an infinite temporal receptive field within a fixed computational budget. By echoing the original bio‐mimetic design of neural networks, which draws inspiration from biological neural propagation, our method naturally satisfies the stringent requirements for efficiency and performance in clinical applications.

While comprehensive scene understanding provides the “what” and “where” of the operative context, robust procedure‐spanning autonomy ultimately depends on the “why” that governs expert decisions. This differs from episodic sub‐tasks autonomy, where black‐box methods like reinforcement learning often succeed without explicit reasoning. We therefore treat expert gaze behavior not merely as a supervision signal, but as a window into the reasoning processes that experts employ during surgery. By systematically mining multi‐level eye‐tracking data, we identified structured intraoperative attention behaviors that are seldom articulated in existing literature. Importantly, we translate these latent patterns into machine‐interpretable formulations that drive autonomous actions, making each view assistance decision traceable to clinically meaningful cues. Such an expertise transfer framework ensures a transparent decision‐making chain, rendering it inherently suitable for autonomous assistance in surgeon‐collaborative scenarios.

The clinical translation of this trustworthy autonomy yields multifaceted value across the surgical ecosystem. For the surgeon, our method guarantees a high‐quality FoV and eliminates the coordination overhead associated with human assistants. This ensures continuous operative momentum and allows surgeons to channel their full attention into operative maneuvers. For the patient, our method holds the potential to mitigate the risk of errors associated with suboptimal visualization and to shorten operative time, which could enhance patient safety and outcomes. At the institutional level, autonomous assistance alleviates the dependency on surgical staff. It further liberates senior assistants from this auxiliary task, allowing them to lead complex procedures and thereby optimizing the utilization of high‐value medical expertise. Collectively, by synergizing these advantages, our framework enhances overall surgical performance and drives holistic operating room efficiency. Furthermore, the contribution of this work extends to long‐term systemic improvements. Beyond autonomous assistance, the comprehensive surgical scene information provided by HGPNet and the characterization of visual attention behaviors hold significant potential as tools for surgical education and quality assessment. This enables novice surgeons to refine their skills by benchmarking their own gaze patterns and operative sequences against expert standards. Furthermore, by abstracting decision‐making from specific hardware constraints, our framework enables seamless adaptation to diverse downstream robotic platforms and telesurgery scenarios. Ultimately, our framework is grounded in the intrinsic nature of surgical tasks and expert decision paths, allowing traceable decision‐making and robust performance to emerge as natural consequences. Such a nature‐aligned paradigm provides a promising pathway for facilitating clinical task autonomy.

Despite these promising results, several limitations motivate our future work. Regarding clinical validation, the current in vivo evaluation is limited in scale. To make a definitive case for patient and institutional benefits, we plan to conduct large‐scale randomized controlled trials encompassing a wider range of subjects and procedures. These trials will incorporate comprehensive cost‐benefit analyzes and expand evaluation metrics to clinical endpoints, such as complication rates and postoperative recovery profiles. At the algorithmic level, scaling our expertise transfer paradigm to new procedures currently requires manual knowledge elicitation. We aim to address this by developing reusable decision primitives. Concurrently, to handle off‐nominal conditions observed in non‐expert trials, we plan to incorporate uncertainty‐aware gating and robust human override protocols. Lastly, toward system‐level deployment, we plan to integrate autonomous lens cleaning capabilities and extend our framework to higher‐level assistance functions to further reduce operative time and enhance procedural fluency. Ultimately, integrating these advancements with clinical‐grade surgical robots will ensure full compatibility with standard operating room workflows, paving the way for clinical applications.

## 4 Methods

### 4.1 Hierarchical Granularity Perception Network

HGPNet jointly predicts pixel‐level segmentation, fine‐grained action recognition, and sequence‐level workflow state. These outputs provide downstream assistance decision‐making with a coherent information hierarchy, spanning local operative details and global semantic context. To this end, HGPNet is architected around reconciling the heterogeneous representational demands across these granularities while satisfying the strict real‐time latency constraints required for clinical deployment.

As illustrated in Figure 2a, HGPNet features a meticulously designed architecture comprising shared feature extraction, task‐specialized processing, and cross‐granularity interaction. Regarding shared feature extraction, a Mask2Former‐based backbone [52] is employed to capture universal representations compatible with diverse granularities, thereby minimizing redundant computation. For task‐specialized processing, we devised two novel modules tailored to the distinct inductive biases of different outputs. Specifically, our motion interaction learning (MIL) mechanism enhances instance‐level segmentation by explicitly modeling the bidirectional dynamics between instruments and tissues. Conversely, our hierarchical temporal memory bank (HTMB) mechanism provides multi‐scale temporal context for sequence‐level recognition, encompassing phases, steps, and action triplets. Finally, to bridge these representations, we construct explicit cross‐granularity pathways that enable reciprocal exchange of complementary cues between pixel‐level dynamics captured by MIL and workflow‐level semantics modeled by HTMB (see Section S8). The resulting segmentation masks are further converted into standardized surgical‐region reference points via topology aware post‐processing [48], which provides stable spatial targets for downstream decision‐making.

#### 4.1.1 Motion Interaction Learning

Surgical dynamics constitute a deterministic paradigm comprising instrument trajectories and tissue deformation linked by causal coupling. Capturing this structured instrument‐tissue coupling provides a strong prior for stabilizing instance‐level perception and improving downstream semantic understanding. To this end, we introduce MIL, as illustrated in Figure S5. This module progressively distills multi‐level motion semantics by transitioning from instance matching and behavioral auto‐encoding to explicit cross‐entity interaction modeling.

To explicitly model this prior, MIL partitions the backbone object tokens into instrument and tissue subsets [53]. Given the permutation‐invariant nature of the backbone output, we employ Hungarian matching to establish inter‐frame correspondence for instruments. Conversely, leveraging the anatomical uniqueness where each tissue class appears as a single instance, we utilize fixed category‐based matching for tissue tokens. On top of these temporally associated tracks, MIL encodes self‐motion patterns for both tools and tissues via temporal self‐attention, formulated as:

(1) $$Z^{\text{inst}}=A_{\text{temp}}\left(X^{\text{inst}}\right),\;Z^{\text{tiss}}=A_{\text{temp}}\left(X^{\text{tiss}}\right)$$

where $A_{\text{temp}}\left(\cdot \right)$ denotes the temporal self‐attention operator, while $Z^{\text{inst}}$ and $Z^{\text{tiss}}$ represent the dynamic motion representations extracted from the static instrument embeddings $X^{\text{inst}}$ and tissue embeddings $X^{\text{tiss}}$, respectively. Consequently, MIL implicitly captures latent motion attributes, such as velocity and acceleration for instruments, and deformation patterns for tissues, directly from the temporal evolution of these tokens.

Moreover, surgeons simultaneously monitor instrument actions and tissue responses, forming a bidirectional perceptual loop that is central to surgical expertise. Drawing inspiration from this, we design a symmetric cross‐attention mechanism to explicitly model the reciprocal interactions between tools and tissues. This mechanism facilitates two parallel information streams:

(2) $$H^{\text{inst}}=B_{\text{inst}\to \text{tiss}}\left(Z^{\text{inst}},Z^{\text{tiss}}\right),\;H^{\text{tiss}}=B_{\text{tiss}\to \text{inst}}\left(Z^{\text{tiss}},Z^{\text{inst}}\right)$$

where B denotes the cross‐attention interaction operator. Specifically, the instrument‐to‐tissue interaction $H^{\text{inst}}$ captures how tool motion perturbs tissue, while the tissue‐to‐instrument interaction $H^{\text{tiss}}$ enables inverse reasoning to deduce which tool motion best explains the observed tissue responses.

These representations subsequently undergo non‐linear transformation and feature refinement via a feed‐forward network, yielding the refined embeddings ${\tilde{H}}^{\text{inst}}$ and ${\tilde{H}}^{\text{tiss}}$. Finally, we concatenate these features to construct the comprehensive motion‐interaction embeddings:

(3) $$V_{\text{motion}}=\text{Concat}\left({\tilde{H}}^{\text{inst}},{\tilde{H}}^{\text{tiss}}\right)$$

#### 4.1.2 Hierarchical Temporal Memory Bank

Temporal dependency differs substantially across granularity levels. Surgical phase recognition depends on long‐horizon context spanning minutes, action‐triplet recognition depends on short‐lived interactions over seconds, and surgical step recognition lies between these extremes. This hierarchical demand parallels the human information processing mechanism described by the Atkinson–Shiffrin memory model [54], which posits a sequential system comprising sensory, short‐term, and long‐term memory.

In this model, sensory memory briefly holds raw environmental input, which is then processed into short‐term memory and selectively consolidated into long‐term storage. Inspired by this architecture, we design the hierarchical temporal memory bank (HTMB) mechanism. Specifically, single‐frame outputs are defined as instantaneous current features $F_{\text{curr}}$. These tokens are buffered in a short‐term memory bank and processed to yield short‐term features $F_{\text{short}}$. Hierarchically, these short‐term features are subsequently accumulated in a long‐term memory bank, where they are further integrated to construct the long‐term representation $F_{\text{long}}$:

(4) $$F_{\text{curr}}=f_{\theta }\left(X\right),\;F_{\text{short}}=A_{S}\left(F_{\text{curr}}\right),\;F_{\text{long}}=A_{L}\left(F_{\text{short}}\right)$$

where $f_{\theta }\left(\cdot \right)$ denotes the shared backbone, $A_{S}\left(\cdot \right)$ denotes the short‐term aggregation operator, and $A_{L}\left(\cdot \right)$ denotes the long‐term aggregation operator.

Given the limited temporal scope of $F_{\text{short}}$, updating the short‐term memory bank via a standard First‐In‐First‐Out (FIFO) queue is sufficient. However, maintaining $F_{\text{long}}$ presents a distinct challenge. The prevailing approach in the domain extends the temporal receptive field simply by increasing the capacity $T_{L}$ of the long‐term memory bank under a FIFO policy. This naive extension suffers from two critical limitations. First, compute and memory costs often scale super‐linearly (e.g., quadratically for attention) with sequence length. Second, indiscriminately retaining large amounts of redundant history, such as completed surgical phases, can introduce noise that interferes with current decision‐making.

In contrast, the human memory system achieves continuous knowledge adaptation under finite neural resources. During the acquisition of new knowledge, correlated neurons co‐activate, establishing and strengthening synaptic connections. Conversely, synaptic pruning eliminates connections corresponding to memories that remain inactive for extended periods, signaling their obsolescence in the current environment. This dynamic mechanism enables the brain to continuously optimize its knowledge base, retaining high‐value information. Drawing inspiration from this, we propose an adaptive update mechanism for the long‐term memory bank. By integrating fusion (mimicking reinforcement) and forgetting (mimicking pruning), we achieve a near infinite temporal receptive field while maintaining a fixed memory capacity.

As illustrated in Figure S6, we first compute the cosine similarity between adjacent feature pairs within the long‐term memory bank:

(5) $$\text{sim}\left(f_{i},f_{j}\right)=\frac{f_{i}\cdot f_{j}}{∥f_{i}∥∥f_{j}∥}$$

Subsequently, we identify the pairs exhibiting the maximum and minimum similarity scores, representing the most redundant and the most isolated feature combinations, respectively. Based on these extremes, we formulate a decision criterion to determine the optimal update strategy:

(6) $$\Delta_{\text{sim}}={\text{sim}}_{\min}-\left(1-{\text{sim}}_{\max}\right)$$

where ${\text{sim}}_{\min}$ and ${\text{sim}}_{\max}$ denote the minimum and maximum similarity values. When $\Delta_{\text{sim}}>0$, it implies that the redundancy characterized by ${\text{sim}}_{\max}$ is excessive. Consequently, the corresponding high‐similarity pair is merged into a single prototype via average pooling. When $\Delta_{\text{sim}}<0$, it indicates the presence of an isolated feature significantly distinct from the context. In this scenario, within the minimum similarity pair, the feature with the earlier timestamp is excised from the bank. Clinically, such pruned features typically correspond to concluded surgical phases or non‐critical events like extracorporeal lens cleaning. Removing these elements refines the memory bank's focus on the current core task, preventing interference from irrelevant historical noise.

Through this mechanism, the long‐term memory bank evolves from a static buffer into a dynamic system capable of active learning and forgetting. This ensures the continuous tracking and distillation of high‐value temporal context across the entire video sequence while maintaining strict computational efficiency.

### 4.2 Modeling Visual Attention Behaviors

Adopting a Bayesian inference formulation [55], We formulate surgeon visual attention behaviors as a probabilistic inference process that synthesizes triplet‐conditioned dynamics (Behavior 1) with phase‐conditioned constraints (Behavior 2). Formally, we model the probability of a spatial location $P_{L}$ receiving attention as a posterior distribution, conditioned on the recognized surgical action triplets $C_{t}$ and the surgical phase $\Phi_{k}$:

(7) $$p\left(P_{L}|C_{t},\Phi_{k}\right)\propto p\left(C_{t}|P_{L}\right)\cdot p\left(P_{L}|\Phi_{k}\right)$$

where the likelihood $p(C_{t}|P_{L})$ functions as an observation model capturing action‐driven prioritization, while the prior $p(P_{L}|\Phi_{k})$ serves as a spatial regularizer reflecting phase‐dependent focal anchoring.

To model Behavior 1, the likelihood $p(C_{t}|P_{L})$ quantifies how ongoing operative interactions competitively attract attention. We assign a base utility to each active triplet derived from expert fixation statistics, incorporating a nonlinear competition term to suppress secondary actions when a dominant triplet emerges. This utility‐based formulation mimics the focus scan alternation observed in expert gaze. These utilities are projected into the image space via kernels centered on interaction regions, yielding a normalized attention‐evidence map (see Section S9).

To model Behavior 2, the prior $p(P_{L}|\Phi_{k})$ captures long‐horizon attentional anchoring governed by procedural goals. Attentional demands vary significantly across phases, ranging from focused scrutiny during manipulation to broad monitoring during exposure. We encode this dependency using a phase‐specific Gaussian prior:

(8) $$p\left(P_{L}|\Phi_{k}\right)\propto \exp\left(-\frac{∥P_{L}-P_{\text{core}}{\left(k\right)∥}^{2}}{2\sigma_{k}^{2}}\right)$$

where $P_{\text{core}}\left(k\right)$ denotes the phase‐specific attentional center and $\sigma_{k}$ controls the spatial dispersion, with smaller values representing concentrated focus and larger values representing distributed monitoring.

Finally, fusing the likelihood and prior yields the normalized posterior attention distribution:

(9) $$p\left(P_{L}|C_{t},\Phi_{k}\right)=\frac{p\left(C_{t}|P_{L}\right)\cdot p\left(P_{L}|\Phi_{k}\right)}{\sum_{P_{L}^{\prime }\in L}p\left(C_{t}|P_{L}^{\prime }\right)\cdot p\left(P_{L}^{\prime }|\Phi_{k}\right)}$$

where L represents the set of all image locations. The resulting posterior offers an interpretable attention map, where the mode $P_{L}^{*}\left(t\right)$ identifies the predicted focus of surgeon attention. This distribution serves as the input for the view assistance policy.

### 4.3 Autonomous View Assistance Policy

In this section, we explicitly model Behavior 3, which is formalized as the view assistance policy. Leveraging the posterior attention distribution derived above, this modeling process reduces to a decision‐making task: determining whether to execute a re‐centering action toward the predicted attention peak $P_{L}^{*}\left(t\right)$ to align with the surgeon's visual intent. From an optimization perspective, we formulate this as a utility maximization problem. We construct a utility function designed to quantify and compare the expected payoffs of executing an assistance action vs. maintaining a wait state, formulated as:

(10) $$a^{*}\left(t\right)=\arg\max_{a\in A}J\left(a|t\right)$$

where $A=\{a_{\text{assist}},a_{\text{wait}}\}$ denotes the discrete action space comprising the assistance and wait primitives. The objective function J(a∣t) quantifies the net utility of executing action a at time t, formulated as:

(11) $$J\left(a|t\right)=E_{\text{view}}\left(a|t\right)\cdot E_{\text{trend}}\left(a|t\right)-C_{\text{cost}}\left(a|t\right)$$

Here, $E_{\text{view}}\left(a|t\right)$ represents the immediate gain derived from action a, $E_{\text{trend}}\left(a|t\right)$ quantifies the trend value coefficient yielded by action a, and $C_{\text{cost}}\left(a|t\right)$ imposes a penalty term reflecting the cost associated with the action itself.

The immediate gain term $E_{\text{view}}$ quantifies the expected improvement in visual informativeness resulting from the candidate action. To formalize this metric, we first define a spatial weighting function that assigns higher significance to regions near the field of view (FoV) center, decaying progressively toward the periphery. By integrating the posterior attention distribution $p(P_{L}|C_{t},\Phi_{k})$ with this centrality weight, we obtain a global visual informativeness score. Consequently, $E_{\text{view}}$ is calculated as the differential improvement in this score between the post‐action and pre‐action states (see Section S10.1).

While immediate gain quantifies the current offset, an assistance action becomes redundant if the surgeon attention is predicted to revert toward the current FoV center in the near future. Therefore, we define the trend value coefficient $E_{\text{trend}}$ to evaluate whether a candidate action aligns with future attentional dynamics. Specifically, we estimate the temporal evolution of the attention peak $P_{L}^{*}\left(t\right)$ using an adaptive Kalman filter. Candidate actions are then assessed based on their directional alignment and spatial proximity to the predicted attentional trajectory. This mechanism suppresses reactive oscillations and promotes anticipatory assistance behavior (see Section S10.2).

Finally, the penalty term $C_{\text{cost}}$ serves as a regularizer to mitigate excessive actuation, thereby enhancing system stability and surgical ergonomics. This term primarily comprises: (1) a *frequency cost*, designed to penalize rapid, successive movements that may induce visual fatigue; and (2) a *deviation tolerance cost*, which discourages adjustments for minor offsets that fall within the surgeon's acceptable workspace. This cost formulation implements a soft suppression of unnecessary view assistance rather than a hard threshold (see Section S10.3).

To realize the personality‐conditioned aspect of Behavior 3, we introduce a parameter modulation mechanism driven by the heterogeneous knowledge graph. While the structure of the utility function remains constant, its governing parameters within the objective function J(a∣t) are dynamically adapted based on the operative context decoded by HGPNet. This allows the policy to seamlessly switch between distinct behavioral profiles, ranging from conservative stability to aggressive responsiveness, without altering the underlying decision logic (see Section S10.4).

### 4.4 Closed‐Loop System Deployment

To transition from autonomous decision logic to real‐time surgical assistance actuation, we implement a closed‐loop deployment architecture. The HGPNet, originally optimized in PyTorch (v1.12), is exported to the ONNX format and deployed within a C++ runtime environment using ONNX Runtime (v1.14) with CUDA (v11.8) acceleration. The deployment system adopts a parallel multi‐threaded architecture to decouple visual processing from robot control. In the perception thread, incoming endoscopic frames are resized to 224×224 and processed via GPU‐accelerated inference. The resulting autonomous decision targets are then asynchronously streamed to a dedicated robot execution thread.

Upon receiving the targets, the system generates physical motion commands. Crucially, this actuation is governed by the RCM constraint typical of MIS, where the endoscope must pivot strictly about the trocar insertion point. Since the collaborative robot lacks a mechanical RCM, we enforce this constraint algorithmically using kinematic control. To translate image‐space targets into physically executable motion, we adopt an image‐based visual servoing (IBVS) controller that minimizes the reprojection error between the current view center and the desired target outputs. The lens velocity ${\dot{X}}_{\text{len}}$ is given by:

(12) $${\dot{X}}_{\text{len}}=-\lambda J_{s}^{†}\left(s_{\text{ext}}-s_{\text{ext}}^{*}\right)$$

where λ is a scalar proportional gain. We use a linear proportional controller to prioritize smooth convergence and stability over pixel‐perfect positioning, which is sufficient for view assistance. $J_{s}^{†}$ denotes the pseudo‐inverse of the image Jacobian mapping differential image feature changes to spatial camera velocities; $s_{\text{ext}}$ and $s_{\text{ext}}^{*}$ are the current and desired feature vectors in the extended image space, respectively. Finally, the task‐space velocity ${\dot{X}}_{\text{len}}$ is mapped to joint velocities through a constrained inverse kinematics solver that enforces the RCM constraint, yielding the final robot motion.

## Conflicts of Interest

The authors declare no conflicts of interest.

## Supporting information


**Supporting File 1**: advs78020‐sup‐0001‐S1.mp4.

**Supporting File 2**: advs78020‐sup‐0002‐SuppMat.docx.

## Data Availability

The data that support the findings of this study are available from the corresponding author upon reasonable request.
