# Supplementary material for: An Expertise Transfer Framework For Autonomous Surgical Assistance
Source: Adv Sci (Weinh). 2026 Sep 28:e78020. Online ahead of print. doi: 10.1002/advs.78020 (PMC13620268; doi:10.1002/advs.78020)
Supplement: Supplementary file 1 [file ADVS-9999-e78020-s001.docx]

# Supporting Information

# An Expertise Transfer Framework for Autonomous Surgical Assistance

*Yuan Gao, Guangdi Chu, Wei Jiao, Yuchuan Zhong, Hang Yuan, Chengjun Li, Jinhua Li, Shuxin Wang, Bo Guan, Jianchang Zhao, Lizhi Pan, Haitao Niu, Jianmin Li*

# 1 | Hierarchical Granularity Dataset

## 1.1 | Data Source and Annotation

Clinical data were collected from two high-volume hospitals with established expertise in urology: the Affiliated Hospital of Qingdao University and the First Affiliated Hospital of Anhui Medical University. The dataset, termed LN-F30, comprises full-length videos from 30 distinct patients undergoing laparoscopic nephrectomy. Excessively long non-informative segments at the beginning and end of each recording were trimmed. All procedures were performed by 10 senior urologic surgeons (mean laparoscopic experience >20 years) following standardized surgical protocols.

To support hierarchical granularity learning, we provide four types of annotations: surgical phases, surgical steps, surgical action triplets, and surgical scene segmentation. Annotations across all granularities were designed to be tightly aligned on a shared temporal axis, enabling direct correspondence across tasks. The final dataset contains 102,284 phase labels, 102,284 step labels, 28,106 action triplet labels, and 8,435 scene segmentation masks.

For surgical phase and surgical step annotation, we convened an expert panel of three urologists who independently annotated the start and end timestamps of each phase and step throughout every video. As transitions between phases/steps are inherently gradual in real surgical workflows, boundary timestamps annotated by different experts may differ slightly. We therefore adopted a majority voting scheme to determine the final temporal boundaries. After temporal boundaries were finalized, all videos were downsampled to 1 frame per second (1 FPS), and phase/step labels were generated for each frame by mapping the voted timestamps to the downsampled timeline.

For surgical action triplet annotation, we used an interval-based sampling strategy to balance annotation cost and label quality. Specifically, we sampled at 16-second intervals and annotated the final continuous 3-second clip within each interval. This short-term contextual clip helps annotators capture temporally coherent surgical actions and reduces ambiguity that may arise from isolated single-frame labeling. However, certain clinically important action triplets occur primarily within short-duration, procedure-specific steps, and uniform interval sampling may lead to insufficient examples for these rare classes. To mitigate this class imbalance, for steps that were brief yet clinically critical, we shortened the sampling interval to 8 seconds, thereby increasing coverage of rare but important triplet instances and improving the overall label distribution.

For scene segmentation, we followed a similar sampling principle. We selected one image every 16 seconds, corresponding to the last frame of each interval, for pixel-level annotation. For the same short-duration but clinically critical steps described above, the sampling interval was likewise reduced to 8 seconds to ensure adequate coverage of key scenes. Segmentation annotations were produced using the open-source tool LabelMe, and the resulting masks were post-processed to fill small gaps between adjacent instance contours, improving spatial completeness and boundary consistency. The annotation process was conducted by 20 medically trained annotators who received systematic training in surgical knowledge and procedural workflows. All annotations were subsequently reviewed and corrected by expert urologists.

## 1.2 | Annotation Design

From a clinical perspective, some anatomical structures differ in physiological function and morphology, yet surgeons handle them in highly similar ways during laparoscopic procedures, implying nearly identical requirements for downstream autonomous assistance. For example, the renal artery, renal vein, and ureter are all transected following a similar sequence: dissection from surrounding tissues, clipping to occlude flow, and cutting to complete transection. For autonomous view assistance, these steps consistently require the tubular structure to remain stably centered in the field of view (FoV). Meanwhile, these tubular structures often exhibit highly similar visual appearances (e.g., shape, color, and texture) under laparoscopic imaging, making fine-grained discrimination unreliable and prone to confusion. Therefore, to reduce unnecessary class ambiguity and better serve downstream autonomous assistance, we merged anatomically distinct but operationally similar structures into unified categories. The final compressed category set is shown in Figure S1.

**Figure S1.** Illustration of the final compressed category set in the Hierarchical Granularity Dataset. Surgical action triplets are defined by instrument-verb-target combinations. For surgical scene segmentation, category definitions follow the instrument and target categories used in the surgical action triplets.

# 2 | Evaluation Metrics for HGPNet

Let $C$ denote the set of classes for a given task and $N$ the number of evaluated samples. We use the standard definitions of true positives (TP), false positives (FP), false negatives (FN), and true negatives (TN). Unless otherwise stated, metrics for multi-class recognition are computed in a one-vs-rest manner for each class and then averaged across classes (macro average).

Accuracy is defined as:

$$\text{Acc}=\frac{1}{N}\sum_{i=1}^{N} \mathbf{1} \left(\hat{y}_{i}=y_{i}\right)$$

For each class *c*, precision and recall are defined as:

$$\text{Prec}_{c}=\frac{\text{TP}_{c}}{\text{TP}_{c}+\text{FP}_{c}},\text{ Rec}_{c}=\frac{\text{TP}_{c}}{\text{TP}_{c}+\text{FN}_{c}}$$

The Jaccard index is computed per class as:

$$\text{Jac}_{c}=\frac{\text{TP}_{c}}{\text{TP}_{c}+\text{FP}_{c}+\text{FN}_{c}}$$

For each triplet class *c*, we compute the average precision as the area under the precision-recall curve obtained by varying the decision threshold on the predicted confidence scores. Mean average precision (mAP) is then defined as：

$$\text{mAP}=\frac{1}{|C|}\sum_{c\in C} \text{AP}_{c}$$

For surgical scene segmentation, we report mean intersection-over-union (mIoU), mean Dice coefficient (mDice), and mean boundary F1-score (mBF1). For each segmentation class *c*, IoU is defined as:

$$\text{IoU}_{c}=\frac{|P_{c}\cap G_{c}|}{|P_{c}\cup G_{c}|}$$

where $P_{c}$ and $G_{c}$ denote the predicted and ground-truth pixel sets of class *c*, respectively. mIoU is obtained by averaging $\text{IoU}_{c}$ over classes. The Dice coefficient is defined per class as：

$$\text{Dice}_{c}=\frac{2|P_{c}\cap G_{c}|}{|P_{c}|+|G_{c}|}$$

and mDice is computed by averaging over classes. To assess boundary quality, we additionally compute the boundary F1-score by matching predicted and ground-truth boundaries and calculating precision and recall on boundary pixels; mBF1 denotes the class-averaged boundary F1-score.

# 3 | Analysis of Failure Cases in Surgical Phase Recognition

Representative failure cases are highlighted in Figure S2. When the FoV is obscured by blood contamination or surgical smoke, the procedure may temporarily enter the idle phase because the surgeon pauses to restore visibility. However, residual visual cues in the frames can still prompt HGPNet to predict a neighboring active phase, resulting in transient misclassifications. In the final stage of laparoscopic nephrectomy (LN), the surgeon typically performs hemostasis and irrigation of the resection bed after complete kidney removal. However, whether the operative field has been sufficiently cleaned does not have an objective visual criterion and is often determined by the surgeon’s judgment. Consequently, when a small amount of residual saline or blood remains but the surgeon decides that no further intervention is necessary and transitions to the idle phase, the model may still associate residual fluids with ongoing surgical activity and misclassify the segment as the kidney mobilization phase. In addition, phase transitions are inherently gradual, leading to ambiguous boundaries. HGPNet can exhibit confusion and short-lived label oscillations in these transitional regions.

**Figure S2.** Frame-level visualization of typical failure cases in surgical phase recognition by HGPNet.

# 4 | Illustration of dissection patterns lead to different view assistance strategies

**Figure S3.** Illustration of dissection patterns lead to different view assistance strategies. Ultrasonic scalpel dissection typically produces a localized action region. When this compact dissection region drifts toward the edge of the FoV, it is likely to trigger a view assistance action to recenter the operative area. In contrast, irrigator-based blunt dissection spans a broader region. Even when part of the dissection activity approaches the FoV boundary, sufficient operative context often remains visible, and a view-assistance action may not be triggered.

# 5 | Assessment of system generalization in a simulated laparoscopic cholecystectomy task

Figure S4. Overview of the standardized LC workflow used to probe cross-procedure transfer, consisting of liver retraction, gallbladder bed dissection, and cystic duct isolation.

# 6 | Details for Quantifying Physical Fatigue and Cognitive Load

## 6.1 | Surface Electromyography (sEMG) Protocol

**Instrumentation and Subject Configuration**

Surface electromyography (sEMG) data were acquired utilizing the Delsys Trigno wireless system (Delsys Inc., Boston, MA, USA). The wireless architecture was selected to preserve the natural kinematic chain of the surgeon's movements. Four healthy, right-handed male volunteers (age range: 24–37 years) with no history of upper-limb neuromuscular disorders participated in the study. These participants included the primary surgeon and the three surgical assistants from the simulation trials. Following standardized skin preparation—consisting of hair removal and exfoliation with 75% isopropyl alcohol to minimize skin-electrode impedance—six wireless sensors were symmetrically deployed. Based on SENIAM recommendations, sensors were placed over the bellies of the Flexor Digitorum Superficialis (FDS), Flexor Carpi Radialis (FCR), and Abductor Pollicis Brevis (APB). Although two additional channels were initially placed to monitor wrist extension, they were excluded from final analysis due to insufficient activation levels. All signals were sampled at 2000 Hz.

**Fatigue Induction and Experimental Paradigm**

A standardized Maximum Voluntary Contraction (MVC) paradigm was implemented to induce and evaluate localized muscle fatigue. The procedure involved four phases: baseline pre-test, task execution, fatigue post-test, and a controlled washout period. In the pre-test, participants performed three independent 5-second isometric contractions (wrist flexion, metacarpophalangeal flexion, and thumb grasping), each separated by a 5-second rest. Subsequently, a 3-minute simulated cholecystectomy was performed following the established surgical workflow. A post-task MVC sequence, identical to the pre-test, was executed immediately to capture the acute fatigue state. To prevent cumulative fatigue bias, an ABBA crossover design was employed, with washout periods determined by both the subject’s perceived recovery and the stabilization of the sEMG baseline.

**Feature Extraction and Fatigue Quantification**

Raw sEMG signals were processed using sliding window envelopes to isolate stable core segments. In the time domain, Root Mean Square (RMS) was calculated to quantify motor unit recruitment and sustained muscular tension. Frequency-domain analysis utilized the Welch method to estimate Power Spectral Density (PSD). Two primary frequency metrics, Median Frequency (MDF) and Mean Power Frequency (MPF), were derived to evaluate spectral shifts. A decrease in these frequency components (leftward spectral shift) was utilized as a physiological marker of lactic acid accumulation and the resultant decline in muscle fiber conduction velocity.

## 6.2 | Electroencephalography (EEG) Analytics

**Data Acquisition and Synchronization**

Continuous 64-channel EEG recordings were obtained via a BrainVision actiCHamp Plus amplifier using ActiCAP slim active electrodes (Brain Products GmbH, Munich, Germany). The montage followed the extended 10-20 international system, with all channels referenced to FCz online and the ground placed at the forehead. Electrode impedances were maintained below 10 kΩ, and the sampling rate was set to 1000 Hz. Due to the high-density configuration and prolonged preparation requirements, this assessment was conducted as an intensive dual-modal case study on the primary surgeon, synchronized with the EMG recordings and trial sequences. A 1-minute resting-state baseline was recorded prior to the trials for normalization.

**Signal Preprocessing Pipeline**

Offline preprocessing was conducted in the EEGLAB environment. Power-line interference was mitigated using a 50 Hz notch filter. Channel integrity was visually inspected, and abnormal leads were reconstructed using spherical spline interpolation. Following downsampling to 250 Hz and band-pass filtering (0.1–30 Hz, FIR filter), the data were re-referenced to the common average. Independent Component Analysis (ICA) was subsequently performed to identify and suppress non-neural artifacts (e.g., ocular and myogenic noise).

**Cognitive Load Metrics**

Following the methodology described by Di Flumeri et al., the task data were segmented into 2-second non-overlapping epochs. For each epoch, PSD was estimated via the Welch method. Two specific neural markers were isolated: absolute Theta power (4–8 Hz) at the frontal Fz channel and absolute Alpha power (8–12 Hz) at the parietal Pz channel. The Theta/Alpha Ratio (TAR) was then calculated for each epoch as a robust physiological index of mental workload and executive control demands.

# 7 | Illustration of Motion Interaction Learning (MIL)

Figure S5. Illustration of Motion Interaction Learning.

# 8 | Illustration of the Adaptive Update Mechanism in Hierarchical Temporal Memory Bank (HTMB) Mechanism.

Figure S6. Illustration of our adaptive update mechanism in hierarchical temporal memory bank mechanism.

# 9 | Cross-granularity Information Interaction in HGPNet

To foster information exchange across granularities, we inject explicit motion-interaction representations from MIL into the hierarchical temporal features from HTMM, leveraging motion priors to purposefully refine the temporal context.

As shown in Figure S7, we first perform max-pooling along the instance dimension of the instrument and tissue interaction embeddings. This distills the most salient global motion characteristics for the current frame, yielding the global instrument representation $\mathbf{m}_{\text{inst}}$ and global tissue representation $\mathbf{m}_{\text{tiss}}$.

Subsequently, we design a motion calibration operator $\Psi$ to target and enhance the instantaneous, short-term, and long-term temporal features $\mathbf{F}_{\tau}$ conditioned on these global motion priors. The process is formulated as:

$$\widehat{\mathbf{F}}_{\tau}=\Psi_{\mathbf{m}_{\text{inst}},\mathbf{m}_{\text{tiss}}}(\mathbf{F}_{\tau}),\text{ }\tau\in\{\text{curr},\text{short},\text{long}\}$$

where, the operator $\Psi$ comprises residual connections and cascaded context attention functions $\Phi$, defined formally as:

$$\Psi_{\mathbf{m}_{\text{inst}},\mathbf{m}_{\text{tiss}}}\triangleq\mathbf{F}_{\tau}+\Phi\left(\Phi\left(\mathbf{F}_{\tau},\mathbf{m}_{\text{inst}}\right),\mathbf{m}_{\text{tiss}}\right)$$

where $\Phi$ adopts a standard cross attention module where temporal features serve as queries, while motion contexts serve as both keys and values:

$$\Phi\triangleq\text{CrossAttn}\left(\mathbf{W}_{\tau}^{Q}\mathbf{F}_{\tau},\mathbf{W}_{\tau}^{K}\mathbf{m}_{i},\mathbf{W}_{\tau}^{V}\mathbf{m}_{i}\right),\text{ }i\in\{\text{curr},\text{tiss}\}$$

Through this cascade, the inner $\Phi$ recalibrates temporal memory using context regarding instrument dynamics, while the outer $\Phi$ further refines this representation based on tissue response. Crucially, residual connections are enforced at each stage to preserve original temporal integrity, ensuring the model acquires fine-grained motion semantics without compromising the macroscopic temporal context.

The enhanced instantaneous, short-term, and long-term features are finally concatenated to form the unified temporal representation:

$$\mathbf{F}_{\text{time}}=\text{Concat}\left(\widehat{\mathbf{F}}_{\text{curr}},\widehat{\mathbf{F}}_{\text{short}},\widehat{\mathbf{F}}_{\text{long}}\right)$$

$\mathbf{F}_{\text{time}}$ encapsulates macro-historical context, recent dynamics, and the immediate snapshot—each calibrated by explicit motion cues. This comprehensive feature is fed into three independent prediction heads, which adaptively extract the necessary temporal cues to output final predictions for surgical phases, steps, and action triplets.

Figure S7. Illustration of the cross-granularity information interaction in HGPNet.

# 10 | Visual-attention Behavior 1 Modeling

First, to quantitatively describe the spatial influence of recognized triplets on the surgical FoV, we construct an instantaneous utility map $U_{\text{map}}(P_{L})$:

$$U_{\text{map}}\left(P_{L}\right)=\sum_{T_{j}\in S_{A}} U_{\text{net}}\left(T_{j}\right)\cdot G\left(P_{L}-P_{j}^{\text{triplet}}\right)$$

where $S_{A}$ denotes the set of currently active triplets, and $P_{j}^{\text{triplet}}$ represents the core operation location of triplet $T_{j}$. $G(\cdot)$ is a kernel function employed to smooth the discrete utility values into the continuous image space. The term $U_{\text{net}}(T_{j})$ represents the Net Utility of triplet $T_{j}$, defined as the difference between its base utility $U_{\text{base}}(T_{j})$ and the cumulative interaction cost:

$$U_{\text{net}}\left(T_{j}\right)=U_{\text{base}}\left(T_{j}\right)-\beta\sum_{j\neq i} C\left(T_{i},T_{j}\right)$$

where $\beta$ is a penalty coefficient regulating the magnitude of interaction costs. The Base Utility $U_{\text{base}}(T_{j})$ quantifies the intrinsic salience of a triplet. This value is calibrated against expert eye-tracking data and is defined as the inverse of the average Euclidean distance between the triplet's core region and the expert's gaze fixation points $P_{m}^{\text{gaze}}$:

$$\begin{matrix} U_{\text{base}}\left(T_{j}\right)=\frac{1}{d_{avg}\left(j\right)+\varepsilon} \\ d_{avg}\left(T_{j}\right)=\frac{1}{M_{j}}\sum_{m=1}^{M_{j}} \left\|P_{m}^{\text{gaze}}-P_{j}^{\text{triplet}}\right\|_{2} \end{matrix}$$

where $\varepsilon$ is a small constant to prevent division by zero, and $M_{j}$ is the number of gaze samples.

The interaction cost function $C(T_{i},T_{j})$ quantifies utility fluctuations arising from inter-triplet competition. In surgical practice, triplets exhibit a distinct functional hierarchy: high-importance triplets capture dominant attention, while secondary triplets are largely ignored. To simulate this polarization, we design $C(T_{i},T_{j})$ as a nonlinear gated saturation function:

$$C\left(T_{i},T_{j}\right)=\tanh\left[\lambda\cdot\text{ReLU}\left(U_{\text{base}}\left(T_{j}\right)-U_{\text{base}}\left(T_{i}\right)-\alpha_{ji}\right)\right]$$

where the $\text{ReLU}(\cdot)$ acts as a gating switch, ensuring the cost mechanism is activated only when the base utility of $T_{j}$ significantly exceeds that of $T_{i}$. The parameter $\alpha_{ji}$ represents the tolerance threshold for utility differences; suppression is triggered only when the disparity surpasses this margin. The $\tanh[\cdot]$ function maps this competitive signal non-linearly to the unit interval [0, 1], modeling an S-curve response where influence grows rapidly from zero to saturation. The parameter $\lambda$ controls the sensitivity (slope) of this transition.

Finally, the scalar utility map $U_{\text{map}}(P_{L})$ is transformed into a probabilistic observation likelihood distribution via an exponential mapping:

$$p\left(C_{t}\left|P_{L}\right.\right)\propto\sigma\left(\gamma U_{map}\left(P_{L}\right)-\alpha_{\text{bias}}\right)$$

where $\sigma(\cdot)$ is the Sigmoid function, $\gamma$ is a gain coefficient controlling the model's confidence in the utility map, and $\alpha_{\text{bias}}$ serves as a bias term to adjust global contrast.

# 11 | Details for Autonomous View Assistance Policy

## 11.1 | Immediate Gain Function

To model the non-uniform distribution of visual information intrinsic to foveal vision, we define the local informativeness $\Phi_{\text{view}}$ using a Gaussian kernel centered at the current view center $C_{V}$:

Here, the Gaussian term with scale $\sigma_{V}$ captures the radial decay of acuity, while the indicator function confines the evaluation to the valid field of view $\Omega_{\text{FOV}}$. By integrating this spatial weighting with the surgeon's posterior attention distribution, we derive the global visual informativeness score:

The immediate gain is strictly defined as the differential improvement in this global score between the candidate state $C_{V}^{*}$ and the current state $C_{V}$:

For an assistance primitive $a_{\text{assist}}$, the view re-centers on the predicted attention peak $C_{V}^{*}=P_{L}^{*}(t)$, maximizing the overlap between the high-resolution foveal zone and the high-probability attention region.

## 11.2 | Trend Value Coefficient

Predicting future surgical trends precisely is difficult and resource-intensive, so we opted to employ a Kalman filter to estimate the trajectory of the attention peak. Upon obtaining this estimation, we construct the trend value coefficient by evaluating the trend across two dimensions: directional alignment and spatial sufficiency. First, the trend vector is computed as a time-decayed weighted sum of future displacements over a prediction horizon $T_{p}$, prioritizing immediate dynamics:

$$\vec{d}_{\text{pred}}(t)=\frac{1}{T_{p}}\sum_{\tau=1}^{T_{p}} \gamma^{\tau-1}\left[\hat{P}_{L}^{*}(t+\tau)-P_{L}^{*}(t)\right]$$

where $\gamma<1$ denotes the decay factor, and $\hat{P}_{L}^{*}$ represents the predicted attention peak. We then quantify the alignment between this predicted trend and the proposed assistance vector $\vec{d}_{\text{move}}(t)$ using the cosine similarity coefficient:

$$S_{\text{dc}}(t)=\cos(\vec{d}_{\text{move}}(t),\vec{d}_{\text{pred}}(t))$$

A negative value ($S_{\text{dc}}(t)<0$) implies that the surgeon's gaze trend is naturally reverting toward the current view center, rendering the assistance action potentially redundant. However, directionality alone is inconclusive; if the magnitude of this regression is minimal—leaving the gaze fixed near the periphery—active adjustment remains essential. To address this, we introduce the reversion sufficiency metric to quantify the extent to which the predicted regression resolves the current offset. We define the minimum distance between the predicted attention peak and the view center over the horizon $T_{p}$ as:

$$D_{\text{mp}}=\min_{\tau\in[1,T_{p}]}\text{‖}\hat{P}_{L}^{*}(t+\tau)-C_{V}\text{‖}$$

Consequently, the sufficiency coefficient is formulated as:

$$S_{\text{suf}}(t)=\max\left(0,\frac{D_{\text{cur}}-D_{\text{mp}}}{D_{\text{cur}}}\right)$$

Assistance is unnecessary only when both conditions are met: the direction is correct and the distance is sufficient. Therefore, the final trend value coefficient is defined to suppress actions only under these specific convergence conditions:

## 11.3 | Cost Regularization

The regularization of the assistance policy is governed by the composite penalty term $C_{\text{cost}}(a|t)$, which aggregates a frequency-dependent stability cost and a spatial tolerance cost:

$$C_{\text{cost}}(a|t)=C_{\text{fre}}(a,t)+C_{\text{tol}}(t)$$

To mitigate motion-induced visual fatigue and suppress high-frequency oscillations, we introduce the frequency cost $C_{\text{fre}}$, governed by a temporal state variable $M(t)$. This variable acts as a motion activation monitor, resetting to unity upon movement and decaying exponentially during idle periods. Mathematically, $M(t)$ is recursively defined as:

$$M(t)=\left\{\begin{matrix} 1, & a_{t-1}=a_{\text{assist}} \\ M(t-1)\cdot e^{-\Delta t/\tau}, & a_{t-1}=a_{\text{wait}} \end{matrix}\right.$$

where $\tau$ is the time decay constant. Consequently, the frequency cost is formulated to penalize consecutive actuation:

$$C_{\text{fre}}(a,t)=\left\{\begin{matrix} \lambda_{m}\cdot M(t), & a_{t-1}=a_{\text{move}} \\ 0, & a_{t-1}=a_{\text{wait}} \end{matrix}\right.$$

Here, $\lambda_{m}$ represents the base penalty coefficient. This design ensures that continuous adjustments incur progressively higher costs, effectively enforcing a refractory period that stabilizes the system behavior.

Regarding spatial constraints, we define the deviation tolerance cost to ensure assistance is triggered only when the view deviation exceeds a physiological comfort zone. First, we define a normalized elliptical distance metric $d_{\text{ell}}(P_{L}^{*},C_{V})$ to characterize the offset of the attention peak relative to the view center:

$$d_{\text{ell}}(P_{L}^{*},C_{V})=\sqrt{\frac{(x^{*}-x_{V})^{2}}{r_{x}^{2}}+\frac{(y^{*}-y_{V})^{2}}{r_{y}^{2}}}$$

where $r_{x}$ and $r_{y}$ denote the semi-axes of the elliptical tolerance region. Based on this metric, the tolerance cost function $C_{\text{tol}}(t)$ is constructed as a non-linear soft barrier:

$$C_{\text{tol}}(t)=\tanh\left[\alpha_{1}(1-d_{\text{ell}}^{v_{1}})-\alpha_{2}\sigma(d_{\text{ell}}-1)(d_{\text{ell}}-1)^{v_{2}}\right]$$

The $\tanh(\cdot)$ function confines the output to the $[-1,1]$ interval. This mechanism ensures that as the gaze deviates further from the center (increasing $d_{\text{ell}}$), the cost $C_{\text{tol}}$ decreases, thereby lowering the barrier for actuation and encouraging necessary view assistance.

## 11.4 | Heterogeneous Knowledge Graph

To bridge high-level semantics and low-level control, we constructed a heterogeneous knowledge graph, as illustrated in Figure S8. This architecture defines a dynamic knowledge propagation path that links surgical workflow nodes to physical entities. Crucially, it maps these semantic attributes directly to specific control parameters, thereby enabling the dynamic instantiation of context-aware behavioral policies. Specifically, to operationalize these mappings and instantiate personality-conditioned behavior, we implement a hierarchical parameter inheritance mechanism where the final parameter set $\theta_{\text{tem}}$ is derived via a cascading override function:

$$\theta_{\text{tem}}=\theta_{\text{def}}\oplus\theta_{\text{phase}}\oplus\theta_{\text{triplet}}$$

where $\theta_{\text{def}}$ represents the default baseline parameters, $\theta_{\text{phase}}$ corresponds to phase-specific adjustments, and $\theta_{\text{triplet}}$ denotes granular instance-specific settings. The operator $\oplus$ enforces a priority rule: $\theta_{\text{triplet}}>\theta_{\text{phase}}>\theta_{\text{def}}$. This hierarchy enables the system to exhibit distinct behavioral profiles, ranging from conservative personality to aggressive personality, by simply loading the appropriate parameter set calibrated from the corresponding subset of expert gaze data.

The distinct personalities are realized by modulating the elliptical tolerance region $\{r_{x},r_{y}\}$ and the cost function shape parameters $\{\alpha_{1},\alpha_{2}\}$. These parameters are calibrated from expert eye-tracking data using the empirical rule (68-95-99.7 rule) of normal distributions. As shown in Figure S9, we partition the visual field into three semantic zones: a comfort zone, a tolerance zone (covering ~95%), and a penalty zone. For any given context-specific data subset, we first compute the sample covariance matrix to derive the standard deviations $\sigma_{x}$ and $\sigma_{y}$. The elliptical tolerance boundary is then defined as $r_{x}=1.8\sigma_{x}$ and $r_{y}=1.8\sigma_{y}$, ensuring that the tolerance region adapts to the natural variance of the surgeon's gaze in that specific context.

Subsequently, the shape parameters $\{\alpha_{1},\alpha_{2}\}$ of the cost function are solved analytically to ensure the penalty gradient aligns with these statistical boundaries. We impose four boundary conditions: maximum tolerance at the center ($C_{\text{tol}}(0)=1$), high tolerance at the comfort boundary ($C_{\text{tol}}(d_{68})=0.8$), zero tolerance at the ellipse edge ($C_{\text{tol}}(1)=0$), and a strong penalty at the hard limit ($C_{\text{tol}}(d_{99.7})=-0.95$). Substituting these conditions into the cost formulation yields the unique analytical solution for these parameters. This process effectively transforms the static semantic structure of the graph into dynamic, autonomous behavioral decisions adapted to the real-time operative context.

Figure S8. Schematic diagram of heterogeneous knowledge graph architecture.

Figure S9. Data-driven parameter calibration based on the empirical rule. (a) The 68-95-99.7 rule of the normal distribution. (b) Spatial mapping and zone definition. The visual field is partitioned such that, ideally, $\sim68\%$ of gaze fixations fall within the comfort zone, representing the optimal visual focus. The tolerance zone is defined to encapsulate $\sim95\%$ of the distribution. The tolerance boundary is set at $1.8\sigma$ rather than the standard $2\sigma$. This adjustment compensates for the distributional contraction caused by the system's active re-centering behavior, which naturally reduces the occurrence of extreme peripheral outliers compared to a purely passive observation distribution.

**Table S1.** Comparison with existing surgical phase recognition SOTAs (%).

| Dataset | Methods | Video-level Metric | Phase-level Metric | | | |
| --- | --- | --- | --- | --- | --- | --- |
|  |  | Accuracy | Precision | Recall | Jaccard |  |
| Cholec80 | TeCNO [1] | 88.6 ± 7.8 | 86.5 | 87.6 | 75.1 |  |
|  | Trans-SVNet [2] | 89.1 ± 6.6 | 84.7 | 83.6 | 72.5 |  |
|  | LoViT [3] | 91.5 ± 6.1 | 83.1 | 86.5 | 74.2 |  |
|  | Surgformer [4] | 92.3 ± 6.2 | 87.1 | **87.6** | 77.8 |  |
|  | HGPNet (Ours) | **92.8 ± 5.8** | **87.5** | 86.9 | **78.1** |  |
| AutoLaparo | TeCNO | 77.3 | 66.9 | 61.5 | 49.6 |  |
|  | Trans-SVNet [2] | 78.3 | 68.0 | 62.2 | 50.7 |  |
|  | LoViT [3] | 81.4 ± 7.6 | **85.1** | 65.9 | 55.9 |  |
|  | SkiT [5] | 82.9 ± 6.8 | 81.8 | 70.1 | 59.9 |  |
|  | HGPNet (Ours) | **85.7 ± 7.2** | 83.8 | **73.2** | **63.7** |  |
| LN-F30 | Trans-SVNet [2] | 83.4 ± 7.9 | 78.4 | 73.8 | 70.4 |  |
|  | LoViT [3] | 91.2 ± 6.3 | 85.8 | 81.3 | 77.0 |  |
|  | HGPNet (Ours) | **93.5 ± 6.1** | **87.9** | **84.5** | **79.3** |  |

**Table S2.** Comparison with existing surgical step recognition methods (%).

| Dataset | Methods | Accuracy |
| --- | --- | --- |
| PSI-AVA | TAPIR [6] | 53.3 |
|  | HCT [7] | 57.7 |
|  | HGPNet (Ours) | **61.9** |
| LN-F30 | TAPIR [6] | 82.5 |
|  | HGPNet (Ours) | **88.7** |

**Table S3.** Comparison with the surgical triplet recognition methods (%).

| Methods | Triplet association performance | | |
| --- | --- | --- | --- |
|  | $\text{mAP}_{\text{IV}}$ | $\text{mAP}_{\text{IT}}$ | $\text{mAP}_{\text{IVT}}$ |
| CholecT45 dataset | | | |
| RDV [8] | 39.4 | 39.6 | 32.7 |
| MT4MTL-KD [9] | 38.5 | 43.3 | 38.5 |
| LAM-Large [10] | - | - | 42.1 |
| HGPNet (Ours) | **41.0** | **48.3** | **48.1** |
| LRN-F30 dataset | | | |
| RDV [8] | 69.6 | 61.4 | 58.5 |
| RiT [11] | 72.8 | 73.0 | 69.1 |
| HGPNet (Ours) | **88.0** | **82.5** | **81.3** |

**Table S4.** Comparison with the surgical scene segmentation SOTAs (%).

| EndoVis18 dataset | | | | | | |
| --- | --- | --- | --- | --- | --- | --- |
| Methods | mIOU | Sequence mIoU | | | | mDice |
|  |  | Seq1 | Seq2 | Seq 3 | Seq 4 |  |
| U-Net [12] | 50.7 | 55.6 | 50.5 | 69.7 | 26.8 | 61.5 |
| DeepLab v3+ [13] | 58.8 | 64.1 | 57.0 | 82.3 | 31.6 | 67.3 |
| STswinCL [14] | 63.6 | 67.0 | 63.4 | 83.7 | 40.3 | 72.0 |
| LSKANet [15] | 66.2 | 67.5 | 63.2 | 85.2 | 48.9 | 75.3 |
| HGPNet (Ours) | **68.5** | 68.9 | **65.7** | **87.0** | **52.3** | **77.2** |
| LR-F30 dataset | | | | | | |
| Methods | mIOU | | mDice | | mBF1 | |
| U-Net [12] | 56.4 | | 71.8 | | 62.3 | |
| DeepLab v3+ [13] | 67.0 | | 79.7 | | 76.6 | |
| LSKANet [15] | 73.4 | | 83.3 | | 79.5 | |
| SAM2 [16] | 59.3 | | 73.5 | | 67.3 | |
| SAM2-UNet [17] | 71.6 | | 82.9 | | 74.6 | |
| HGPNet (Ours) | **78.1** | | **86.1** | | **81.1** | |

**Table S5.** Joint-task performance comparison (%).

| Methods | Phase  Accuracy | Step Accuracy | Triplt  mAP | Segmentation  mIoU | |
| --- | --- | --- | --- | --- | --- |
| HGPNet (Single-Task Evaluation) | 93.5 | 88.7 | 81.3 | **78.1** |  |
| HGPNet (Multi-Task Evaluation) | **93.9** | **89.4** | **84.1** | 75.9 |  |
| Trans-SVNet+Rendezvous+U-Net | 81.6 | 73.9 | 58.1 | 56.4 |  |
| TAPIR+LAM-BASE+LSKANet | 90.3 | 81.5 | 73.3 | 73.4 |  |

**Table S6.** Inference performance comparison (%).

| Methods | Mode | | Parameters（M） | Latency（ms） | Memory usage（GB） |
| --- | --- | --- | --- | --- | --- |
| Trans-SVNet+Rendezvous+U-Net | | Serial | 72.7 | 21.1 | 3.4 |
|  |  | Parallel | 72.7 | 12.3 | 5.9 |
| TAPIR+LAM-BASE+LSKANet | | Serial | 296.9 | 69.8 | 11.2 |
|  |  | Parallel | 296.9 | 32.3 | 32.8 |
| HGPNet (Ours) | | - | 165.8 | 26.7 | 12.8 |

**Table S7.** Quantitative comparison of the operating area's spatial distribution between human assistance and our autonomous assistance.

| Metric | Human assistance | Autonomous assistance (Ours) | |  |
| --- | --- | --- | --- | --- |
| Normalized entropy | 0.94 | | 0.89 | |
| Entropy-derived eff. area | 4.41×10^5^ pixels | | 2.28×10^5^ pixels | |
| Herfindahl–Hirschman index (HHI) | 3.17×10^-6^ | | 7.29×10^-6^ | |
| Inverse- HHI eff. Area | 3.16×10^5^ pixels | | 1.37×10^5^ pixels | |
| 90% density mass area | 38.5% | | 23.1% | |

# Reference

[1] T. Czempiel, M. Paschali, M. Keicher, W. Simson, H. Feussner, S. T. Kim, N. Navab, In *International Conference on Medical Image Computing and Computer-Assisted Intervention*. Springer, 2020 343–352.
[2] X. Gao, Y. Jin, Y. Long, Q. Dou, P.-A. Heng, In *International Conference on Medical Image Computing and Computer-Assisted Intervention*. Springer, 2021 593–603.
[3] Y. Liu, M. Boels, L. C. Garcia-Peraza-Herrera, T. Vercauteren, P. Dasgupta, A. Granados, S. Ourselin, *Medical Image Analysis* 2025, 99 103366.
[4] S. Yang, L. Luo, Q. Wang, H. Chen, In *International Conference on Medical Image Computing and Computer-Assisted Intervention*. Springer, 2024 606–616.
[5] Y. Liu, J. Huo, J. Peng, R. Sparks, P. Dasgupta, A. Granados, S. Ourselin, In *Proceedings of the IEEE/CVF International Conference on Computer Vision*. 2023 21074–21084.
[6] N. Valderrama, P. Ruiz Puentes, I. Hern´andez, N. Ayobi, M. Verlyck, J. Santander, J. Caicedo, N. Fern´andez, P. Arbel´aez, In *International Conference on Medical Image Computing and Computer-Assisted Intervention*. Springer, 2022 442–452.
[7] L. Hao, Y. Hu, Y. Yue, L. Wu, H. Fu, J. Duan, J. Liu, *IEEE Transactions on Circuits and Systems for Video Technology* 2024, 2024, 35,4 3134-3145.
[8] C. I. Nwoye, T. Yu, C. Gonzalez, B. Seeliger, P. Mascagni, D. Mutter, J. Marescaux, N. Padoy, *Medical Image Analysis* 2022, 78 102433.
[9] S. Gui, Z. Wang, J. Chen, X. Zhou, C. Zhang, Y. Cao, *IEEE Transactions on Medical Imaging* 2023, 43, 4 1628.
[10] Y. Li, B. Bai, F. Jia, *International Journal of Computer Assisted Radiology and Surgery* 2024, 19, 7 1291.
[11] S. Sharma, C. I. Nwoye, D. Mutter, N. Padoy, *International Journal of Computer Assisted Radiology and Surgery* 2023, 18, 6 1053.
[12] O. Ronneberger, P. Fischer, T. Brox, In *International Conference on Medical Image Computing and Computer-Assisted Intervention*. Springer, 2015 234–241.
[13] L.-C. Chen, G. Papandreou, I. Kokkinos, K. Murphy, A. L. Yuille, *IEEE Transactions on Pattern Analysis and Machine Intelligence* 2017, 40, 4 834.
[14] Y. Jin, Y. Yu, C. Chen, Z. Zhao, P.-A. Heng, D. Stoyanov, *IEEE Transactions on Medical Imaging* 2022, 41, 11 2991.
[15] M. Liu, Y. Han, J. Wang, C. Wang, Y. Wang, E. Meijering, *IEEE Transactions on Medical Imaging* 2023, 43, 4 1308.
[16] N. Ravi, V. Gabeur, Y.-T. Hu, R. Hu, C. Ryali, T. Ma, H. Khedr, R. R¨adle, C. Rolland, L. Gustafson, et al., *arXiv preprint* arXiv:2408.00714 2024.
[17] X. Xiong, Z. Wu, S. Tan, W. Li, F. Tang, Y. Chen, S. Li, J. Ma, G. Li, *arXiv preprint* arXiv:2408.08870 2024.
